# Erythrocyte patch for enhanced B cell depletion therapy

**DOI:** 10.1126/sciadv.aed3138

**Published:** 2026-05-20

**Authors:** Jiaqi Liu, Fengju Wang, Lian Li

**Affiliations:** Key Laboratory of Drug-Targeting and Drug Delivery System of the Education Ministry and Sichuan Province, Sichuan Engineering Laboratory for Plant-Sourced Drug and Sichuan Research Center for Drug Precision Industrial Technology, West China School of Pharmacy, Sichuan University, Chengdu 610041, China.

## Abstract

Dysregulated B cells are central to many hematologic malignancies and autoimmune diseases, but current B cell–targeted therapies often fail to translate binding into effective signaling. Here, we present CD20 EryPatch, a living dynamic cellular patch that co-opts natural erythrocyte processes of morphological and biological changes, enabling a dual mechanism of micrometer-scale mechanotransduction and phagocytic “tag-and-clear” for enhanced B cell depletion. CD20 EryPatches are designed to adhere to B cells and anchor on CD20 receptors across extensive cell surface areas. Their progressive discocyte-to-echinocyte transition provides deformability-driven traction, enabling localized, large-scale CD20 cross-linking that amplifies downstream apoptosis in target B cells. Concomitant biomarker alteration on CD20 EryPatch converts it into an “eat me” tag attached to the B cell, facilitating further clearance by initiating erythrophagocytosis. This approach proves more effective than standard CD20 monoclonal antibodies in models of B cell disorders, including non-Hodgkin lymphoma, systemic lupus erythematosus, and rheumatoid arthritis.

## Teaser

Morphological and biological changes of erythrocyte are harnessed to treat B cell cancer and autoimmune disease.

## INTRODUCTION

Cell surface receptors transduce extracellular cues into intracellular signals, thereby regulating critical cellular behaviors including communication, migration, and apoptosis (*1*–*3*). Typically, receptors cannot function as freely diffusing monomers; instead, ligand-induced assembly into larger, less mobile clusters is obligatory for initiating signaling cascades (*4*). However, many therapeutics bind targets without further manipulating downstream events due to their inability to spatially rearrange these receptors, resulting in transient, low-efficacy responses. For instance, monoclonal antibodies merely targeting CD20 or death receptors fail to induce direct apoptosis unless hyper–cross-linked by multivalent polymers (*5*, *6*), albumins (*7*, *8*), or DNA nanoscaffold (*9*, *10*). Recent studies demonstrate that higher polymer valency in CD20 cross-linking enhances apoptosis, confirming a positive correlation between receptor cross-linking density and therapeutic efficacy (*11*, *12*). In another study using two interactable nanothreads, Nanothread-1 first strings adjacent CXCR4 receptors into nanoclusters, and then Nanothread-2 coils with Nanothread-1 to weave these nanoclusters into a supramolecular network, generating mesoscale CXCR4 assemblies. This hierarchical organization achieves potent CXCR4 antagonism surpassing monovalent and multivalent ligands (*13*, *14*). These findings establish that receptor cross-linking strategies can be broadly applied to drive downstream signaling modulation across diverse targets, with efficacy critically governed by clustering dimensions. Despite these advances, current synthetic platforms for receptor cross-linking remain constrained in size, unable to achieve micrometer-scale clustering that matches the length scale of natural receptor assemblies on target cells, which could leave nanoclusters fragmented and individually scattered across the cell membrane, potentially generating separate effects and asynchronous function (*9*, *11*, *15*). Hence, synchronization of “outside-in” mechanotransduction necessitates a therapy that is biocompatible, transcends the size constraint of existing biomaterials, and enables enlarged-scale coordination of cross-linked receptors over extensive cell surface areas.

**Fig. 1.**
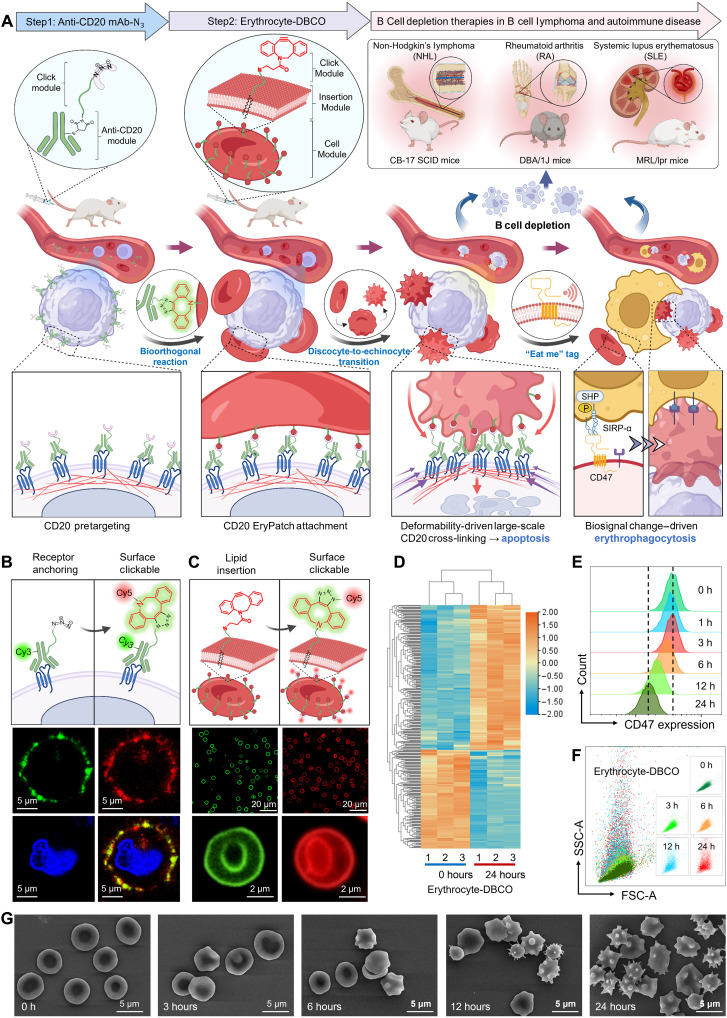
Design and characterization of CD20 EryPatch. (**A**) Illustration showing surface-conformable, interface-deformable CD20 EryPatch for enhanced BCDT through (i) CD20 pretargeting; (ii) attachment to B cell; (iii) discocyte-to-echinocyte transition that drives dynamic interfacial deformation, generating mechanical force for large-scale CD20 cross-linking to amplify downstream apoptosis; and (iv) CD47 down-regulation on EryPatch that initiates erythrophagocytosis. (**B**) Representative confocal laser scanning microscopy (CLSM) images showing bioorthogonal click reaction between bifunctional adaptor [Cy3-labeled anti-CD20 monoclonal antibody (mAb)–N_3_]–pretargeted Raji B cell and DBCO-Cy5. Blue indicates cell nuclei, green indicates Cy3, and red indicates Cy5. Scale bars, 5 μm. (**C**) Representative CLSM images showing erythrocytes with membrane insertion of fluorescein isothiocyanate-(polyethylene glycol)_2000_-1,2-distearoyl-sn-glycero-3-phosphoethanolamine (FITC-PEG_2000_-DSPE) (left panel), and erythrocytes sequentially undergoing DBCO–PEG-2000–DSPE insertion and bioorthogonal click reaction with N_3_-Cy5. Green indicates FITC, and red indicates Cy5. Scale bars, 20 or 2 μm as indicated. Illustrations of (A) to (C) were created in BioRender. Mahaha, M. (2026); https://BioRender.com/0tkfeac. (**D**) Proteomics analyses of Erythrocyte-DBCO 24 hours postinsertion of DBCO–PEG-2000–DSPE. The clustering heatmap reflects the overview of significantly regulated differentially expressed proteins (DEPs) with fold change (FC) > 2 and *P* < 0.05 determined by one-way comparison. (**E** and **F**) Flow cytometry analysis of the eventual alteration in CD47 expression (E) and size/surface roughness (F) on Erythrocyte-DBCO at predetermined time points after the cell surface lipid insertion (*n* = 3). FSC, forward scatter; SSC, side scatter.; h, hours. (**G**) Representative scanning electron microscopy (SEM) images depicting the morphologically discocyte-to-echinocyte transition of EryPatch over time. Scale bars, 5 μm. P, phosphorylation; SIRP-a, Signal regulatory protein alpha; SHP, Src Homology 2 domain-containing Phosphatase.

Accordingly, we developed a surface-conformable, interface-deformable erythrocyte patch (EryPatch) that leverages dynamic biointerface transformations to exert extensive spatial cross-linking of CD20 for enhanced B cell depletion therapy (BCDT). Selective expression of CD20 on pro-B to mature B cells makes it an ideal BCDT target while sparing B cell reconstitution and plasma cell immunosurveillance (*16*). CD20-directed BCDTs are well established for B cell lymphomas and show growing clinical benefits for B cell–mediated autoimmune diseases (*17*). In this study, EryPatch integrates two functional modules: bifunctional adaptors and morphology-transitioning erythrocytes. We propose the following mechanism (Fig. 1A): (i) Bifunctional adaptors pretarget B cells, anchor on CD20, and expose click moieties; (ii) engineered erythrocytes recognize CD20-bound adaptors via bioorthogonal click conjugation, achieving extensive B cell surface attachment; (iii) discocyte-to-echinocyte transition of EryPatch drives dynamic interfacial deformation, generating mechanical force for micrometer-scale CD20 cross-linking to synchronize receptor aggregation and amplify apoptotic signaling; (iv) concomitant CD47 down-regulation on EryPatch converts itself into an “eat me” tag attached to B cell for further clearance via erythrophagocytosis. By co-opting natural erythrocyte processes of morphological and biological changes, we further confirmed the enhanced efficacy of this living dynamic cellular patch in models of B cell disorders, including non-Hodgkin lymphoma (NHL), and autoimmune diseases [rheumatoid arthritis (RA) and systemic lupus erythematosus (SLE)].

## RESULTS

### Biology and morphology change in EryPatch

The bifunctional adaptor [anti-CD20 monoclonal antibody (mAb)–N_3_] comprises a receptor-anchoring module (anti-CD20 mAb) conjugated to azide click module. We synthesized anti-CD20 mAb-N_3_ through controlled partial reduction of the antibody to selectively expose hinge-region thiol groups, followed by site-specific thiol-ene conjugation with maleimide-functionalized N_3_–polyethylene glycol, molecular weight 1000 (PEG-1000)–maleimide (fig. S1A). The average number of conjugated PEG-N_3_ chains per mAb was determined using two complementary methods: (i) by measuring the initial and remaining thiol groups before and after conjugation of N_3_–PEG-1000–maleimide to the thiolated mAb, which indicated approximately 4.9 PEG-N_3_ chains per mAb (fig. S1B); and (ii) by quantifying azido group concentration via a fluorescence-based click reaction with dibenzocyclooctyne-Cy5 (DBCO-Cy5) relative to mAb concentration, which yielded a consistent average of 4.4 chains per mAb (fig. S1C). Reducing sodium dodecyl sulfate-polyacrylamide gel electrophoresis (SDS-PAGE) confirmed successful PEG-1000-N_3_ conjugation, as evidenced by the reduced electrophoretic mobility (upward shift) of both heavy and light chains (fig. S1D). Furthermore, nonreducing SDS-PAGE demonstrated that the modified antibody retained its intact quaternary structure, migrating at ~150 kDa (fig. S1E). Confocal laser scanning microscopy (CLSM) revealed Cy3-labeled anti-CD20 mAb-N_3_ forming ring-pattern surface binding on B cells. Subsequent staining with DBCO-Cy5 showed strong colocalization of Cy3 and Cy5 fluorescence, demonstrating bioorthogonal click reactivity on B cells after anti-CD20 mAb-N_3_ binding (Fig. 1B). Flow cytometry further verified CD20 anchoring (fig. S2) and surface exposure of clickable azide groups (fig. S3) by the bifunctional adaptor anti-CD20 mAb-N_3_.

Morphology-transitioning erythrocytes were engineered by inserting DBCO-functionalized, two-tailed lipids [dibenzocyclooctyne-(polyethylene glycol)2000-1,2-distearoyl-sn-glycero-3-phosphoethanolamine (DBCO–PEG-2000–DSPE)] into red blood cell membranes via hydrophobic interaction, generating Erythrocyte-DBCO presenting complementary DBCO click modules. Initially, Erythrocyte-DBCO had an average size of 4.63 μm (fig. S4). As evident from Fig. 1C, erythrocytes incubated with fluorescein isothiocyanate-(polyethylene glycol)_2000_-1,2-distearoyl-sn-glycero-3-phosphoeth anolamine (FITC-PEG_2000_-DSPE) displayed uniform green fluorescence across cell surfaces, confirming effective lipid insertion for cell surface modification. Consecutive treatment with DBCO–PEG-2000–DSPE followed by N_3_-Cy5 yielded robust cell surface decoration with Cy5, demonstrating successful DBCO modification and specific bioorthogonal reactivity on functionalized Erythrocyte-DBCO. In contrast, erythrocytes exposed only to N_3_-Cy5 showed negligible fluorescence, due to the lack of DBCO groups inserted on cell surface (fig. S5).

Notably, lipid insertion eventually increased surface tension (fig. S6) and reduced membrane fluidity (fig. S7) in Erythrocyte-DBCO, leading to significantly elevated mechanical fragility (fig. S8) and osmotic fragility (fig. S9). Although we did not observe overt hemolysis due to lipid-insertion–based surface modification (fig. S10), this membrane perturbation–induced stress could potentially affect the long-term biological and morphological stability of the engineered erythrocyte. Proteomic analysis revealed substantial alterations in the differentially expressed protein [DEP; fold change (FC) > 2, *P* < 0.05 24 hours post–lipid insertion] (Fig. 1D and fig. S11). These DEP changes were predominantly associated with erythrocyte biological signaling (including homeostasis, senescence, and macrophage-mediated clearance) and structural remodeling (including alterations in the actin cytoskeleton, sarcomere, contractile fibers, myofibrils, cortical cytoskeleton, extracellular matrix, and focal adhesion reorganization) (fig. S12). We specifically validated the progressive decline in CD47 (Fig. 1E and fig. S13) and sialic acid (fig. S14), both of which are key modulators linked to erythrophagocytosis inhibition. Flow cytometry analysis further demonstrated time-dependent increases in Erythrocyte-DBCO surface granularity following lipid insertion (Fig. 1F). Supporting these findings, scanning electron microscopy (SEM) visualization captured the dynamic morphological transition: Erythrocyte-DBCO initially exhibited a typical discocyte morphology (0 hours), began transitioning at 3 hours, progressed through intermediate discocyte-to-echinocyte stages (6 to 12 hours), and ultimately adopted a fully transformed echinocyte-like morphology by 24 hours (Fig. 1G). To reflect the metabolic status of Erythrocyte-DBCO over time, we also evaluated its adenosine 5′-triphosphate (ATP) production, a critical process in these mitochondria-lacking anucleate erythrocyte that relies on anaerobic glycolysis to maintain membrane shape, hemoglobin function, and metabolic homeostasis. Result in fig. S15 revealed an identical trend that Erythrocyte-DBCO displayed a time-dependent decrease in ATP production over a 24-hour period. Collectively, these results demonstrate that the structural and functional integrity of erythrocyte is progressively compromised in a time-dependent manner following DBCO–PEG-2000–DSPE insertion. We attribute this effect primarily to DSPE lipid insertion, not DBCO reactivity, for two reasons: First, the DBCO-azide cycloaddition is a highly selective, catalyst-free bioorthogonal reaction that proceeds rapidly and efficiently in physiological environments of living systems without interfering with biological processes; second, these progressive changes occurred in Erythrocyte-DBCO even in the absence of any azide reaction partner (e.g., anti-CD20 mAb-N_3_). Together, these results highlight a dynamic alteration in both biology and morphology of Erythrocyte-DBCO.

### EryPatch adhesion, deformation, and biointerface transformation

**Fig. 2.**
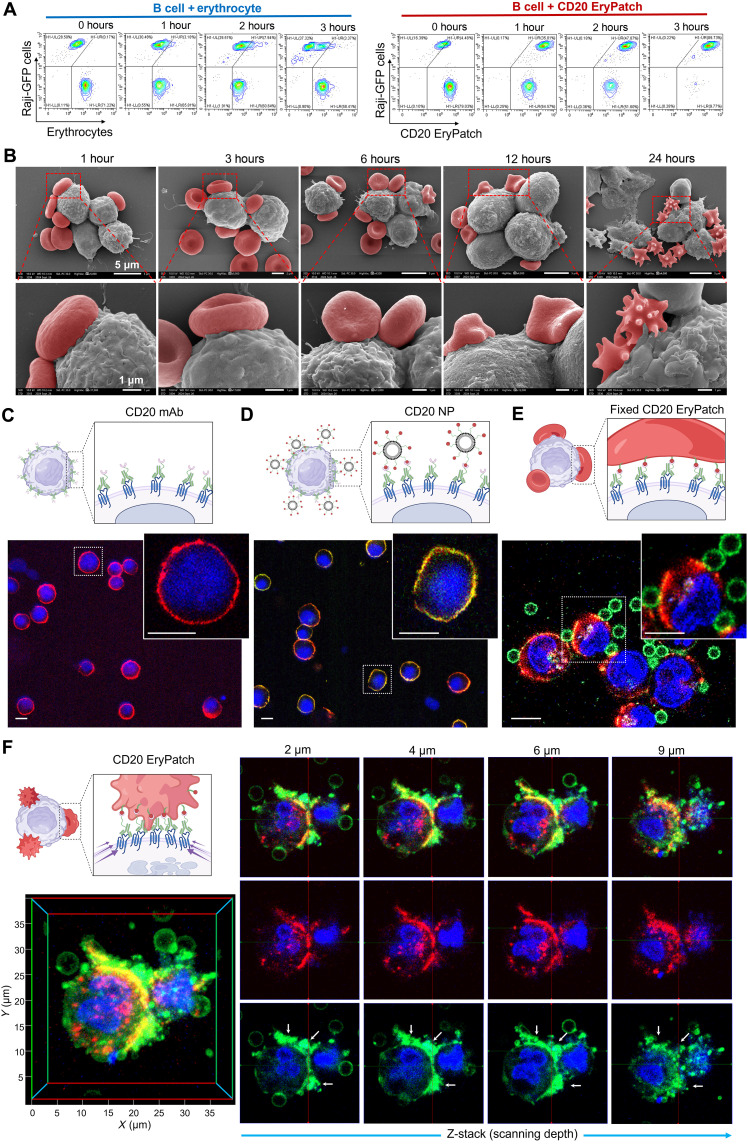
Cell-cell interfacial change after EryPatch attachment and deformation. (**A**) Flow cytometry analysis of erythrocyte or CD20 EryPatch adhesion to Raji B cells. At indicated time points before cell cluster quantification, Raji-GFP were either coincubated with erythrocyte (tracked by red fluorescent signal) or treated with CD20 EryPatch (consecutive anti-CD20 mAb-N_3_ → Erythrocyte-DBCO tracked by red fluorescent signal; 1-hour time lag). (**B**) Representative SEM images depicting sequential cascade of CD20 EryPatch adhesion, discocyte-to-echinocyte deformation, and biointerface transformation upon contact with Raji B cells within 24 hours. Gray indicates Raji B cells, and red indicates CD20 EryPatch. Scale bars, 5 or 1 μm as indicated. (**C** to **E**) Representative CLSM images of Raji B cells treated with CD20 mAb (anti-CD20 mAb-N_3_; 1 hour) (C), CD20 nanoparticle (NP) [anti-CD20 mAb-N_3_ (1 hour) → NP-DBCO (24 hours)] (D), and fixed CD20 EryPatch [anti-CD20 mAb-N_3_ (1 hour) → fixed nondeformed Erythrocyte-DBCO (24 hours)] (E). (**F**) Three-dimensional (3D) construction images of interfacial interaction between Raji B cells and CD20 EryPatch [anti-CD20 mAb-N_3_ (1 hour) → Erythrocyte-DBCO (24 hours)] at different scanning depths. Blue indicates B cell nuclei, red indicates Cy3-labeled anti-CD20 mAb-N_3_, green indicates FITC-labeled NP or Erythrocyte-DBCO using FITC–PEG-2000–DSPE, yellow shows overlay of Cy3 and FITC, and white arrow shows morphogenetic CD20 EryPatch. Scale bars, 10 μm. Illustrations of (C) to (F) were created in BioRender. Mahaha, M. (2026); https://BioRender.com/0tkfeac. The experiments in (A) to (F) were repeated twice independently with similar results.

Having demonstrated the complementary bioorthogonal reactivities on anti-CD20 mAb-N_3_–pretargeted Raji B cells (Fig. 1B) and Erythrocyte-DBCO (Fig. 1C), we proceeded to assess the targeted adhesion capability of CD20 EryPatch (anti-CD20 mAb-N_3_ → Erythrocyte-DBCO; 1-hour interval) to Raji cells. Normally, when green fluorescent protein–expressing Raji cells (Raji-GFP) were mixed with red fluorescently labeled erythrocytes, the two cell populations in flow cytometry scatter plots remained distinctly separated during coincubation, suggesting minimal spontaneous interaction. In notable contrast, as early as 1 hour after EryPatch treatment where anti-CD20 mAb-N_3_–pretargeted Raji-GFP cells were mixed with an excess of Erythrocyte-DBCO, the Raji-GFP cell population entirely shifted to form clusters with Erythrocyte-DBCO, suggesting that nearly all anti-CD20 mAb-N_3_–pretargeted Raji-GFP cells were rapidly attached by at least one Erythrocyte-DBCO. This adhesion process progressed over time, with increasing cluster formation paralleled by unbound Erythrocyte-DBCO population diminishing eventually until complete attachment of CD20 EryPatch to Raji B cells was achieved by 3 hours, evidenced by consolidation into a single dominant cluster population (Fig. 2A). These findings demonstrate that CD20 EryPatch enables rapid, specific, and efficient B cell adhesion through bioorthogonal click chemistry.

SEM imaging (Fig. 2B) further showed that when given the treatment for 1 and 3 hours, Raji cells had multiple discrete CD20 EryPatches attached in the form of discocytes, each occupying micrometer-scale area on Raji cell surface. From 6 to 12 hours, CD20 EryPatches maintained stable adhesion onto Raji cells with conformable cell-cell contact and began undergoing morphological changes, gradually losing their characteristic biconcave disk shape. By 24 hours, the attached CD20 EryPatches had transformed into echinocytes, resulting in biointerface transformation. These results demonstrate that CD20 EryPatches stably and conformally adhere to B cell surfaces while progressively deforming from discocytes to echinocytes.

Having characterized the sequential discocyte-to-echinocyte remodeling of CD20 EryPatch on B cell surfaces, we next evaluated its capacity for spatial rearrangement of CD20-bound mAbs using CLSM. Given that nanoscale polymers and nanoparticles can induce receptor cross-linking (*18*), we prepared DBCO-functionalized nanoparticles (NP-DBCO) via nanoprecipitation using DBCO–PEG-2000–DSPE, poly(lactic-co-glycolic acid) (PLGA), and soy phospholipids. These NP-DBCO exhibited a hydrodynamic diameter of 115.8 ± 1.976 nm, polydispersity index of 0.291, and spherical morphology (fig. S16). For comparison with CD20 EryPatch, CD20 NP was designed as consecutive anti-CD20 mAb-N_3_ → NP-DBCO with 1-hour interval. In addition, fixed CD20 EryPatch was also designed as consecutive anti-CD20 mAb-N_3_ → fixed nondeformed Erythrocyte-DBCO with 1-hour interval. As CD20 is a slowly internalizing receptor (*19*, *20*), Cy3-labeled anti-CD20 mAb exhibited characteristic ring-pattern surface binding on B cells (Fig. 2C). Subsequent multivalent cross-linking through FITC-labeled NP-DBCO binding to the anchored anti-CD20 mAb-N_3_ produced strongly overlapping Cy3/FITC fluorescence signals on Raji cell surfaces. However, rather than showing obvious redistribution of CD20-bound mAb, we only observed diffuse clustering across the membrane, likely due to the small-scale size of the NP-induced nanoclusters (Fig. 2D). In fixed CD20 EryPatch treatment where CD20-bound mAb-N_3_ was cross-linked with large-scale, rigid Erythrocyte-DBCO that had its morphology fixed, Raji cells could be attached by several nondeformable erythrocytes. However, drift in CD20-bound mAb distribution on cell surface remained limited, likely due to the inability of fixed erythrocytes to generate deformability-mediated traction forces for receptor gathering (Fig. 2E). Markedly, treatment with surface-conformable, morphology-deformable CD20 EryPatch resulted in large-scale erythrocyte coverage across Raji cell surfaces, exhibiting irregular fluorescence patterns that aligned with EryPatch deformation. Notably, CD20-bound mAb underwent pronounced redistribution, transitioning from dispersed surface speckles to enlarged punctate aggregates concentrating at the Raji cell–deformed erythrocyte biointerface (Fig. 2F). These findings demonstrate that efficient spatial reorganization of CD20-bound mAb by CD20 EryPatch requires both large-scale coverage and deformability-driven traction.

### Amplified CD20 cross-linking and B cell depletion

**Fig. 3.**
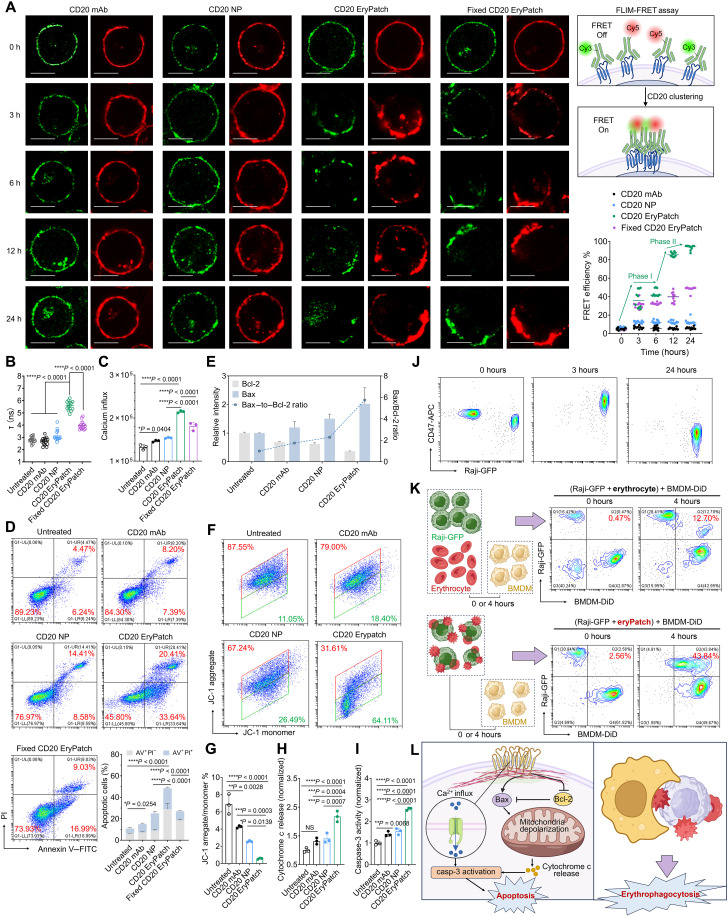
B cell depletion via CD20 EryPatch–induced apoptosis and erythrophagocytosis. (**A**) FLIM-FRET analysis of CD20 cross-linking efficiency. Dual-tagged CD20 receptors on Raji cells via equimolar Cy3- and Cy5-conjugated anti-CD20 mAb-N_3_ were either left untreated (CD20 mAb) or cross-linked with NP-DBCO (CD20 NP), Erythrocyte-DBCO (CD20 EryPatch), or nondeformed Erythrocyte-DBCO (fixed CD20 EryPatch). Green indicates Cy3, and red indicates Cy5. Scale bars, 10 μm. FRET efficiency, reflecting CD20 clustering extent, was calculated on the basis of Cy3 donor lifetimes in the presence and absence of Cy5 acceptor (*n* = 10). (**B**) FLIM quantified changes in Raji cell membrane tension after surface CD20 receptors were bound by CD20 mAb or cross-linked by CD20 NP, CD20 EryPatch, or fixed (nondeformable) CD20 EryPatch using Flipper-TR probe with its fluorescence lifetime (τ) correlating positively with mechanical stress (*n* = 15). (**C** to **I**) Evaluations of downstream signaling manipulation in Raji cells treated with CD20 mAb, CD20 NP, or CD20 EryPatch, including Ca^2+^ influx (C), apoptosis induction (D), Bax/Bcl-2 expression (E), mitochondrial depolarization [(F) and (G)], cytochrome c release (H), and caspase 3 activation (I). NS, not significant. (**J**) Flow cytometry scatter plot representation of anti-CD20 mAb-N_3_–pretargeted Raji-GFP cells and Erythrocyte-DBCO after 0, 3, and 24 hours of mixture, followed by CD47 staining. (**K**) Flow cytometry analysis of phagocytosis of Raji-GFP cells by 1,1′-Dioctadecyl-3,3,3′,3′-tetramethylindodicarbocyanine (DiD)-labeled BMDMs (BMDM-DiD). Following 24 hours premixture of Raji-GFP cells + erythrocytes, or anti-CD20 mAb-N_3_–pretargeted Raji-GFP cells + Erythrocyte-DBCO (Raji-GFP + CD20 EryPatch), stimulated BMDM-DiD were added, allowing for 4 hours of phagocytosis. (**L**) Illustration showing CD20 EryPatch–enhanced B cell depletion via apoptosis and erythrophagocytosis. Created in BioRender. Mahaha, M. (2026); https://BioRender.com/0tkfeac. *n* = 3 in (C) to (K). casp-3, caspase-3. Data are presented as mean ± SD, with statistics calculated via one-way analysis of variance (ANOVA) followed by Tukey’s multiple comparisons. *P < 0.05, **P < 0.01, ***P < 0.001, and ****P < 0.0001.

Using fluorescence lifetime imaging microscopy–Förster resonance energy transfer (FLIM-FRET), we further demonstrated a two-step escalation in CD20 cross-linking induced by CD20 EryPatch (Fig. 3A). Raji cell CD20 receptors were dual labeled with the FRET pair Cy3 (donor) and Cy5 (acceptor) using equimolar Cy3- and Cy5-conjugated anti-CD20 mAb-N_3_. Dual-labeled receptors were either left untreated (CD20 mAb) or cross-linked with NP-DBCO (CD20 NP), Erythrocyte-DBCO (CD20 EryPatch), or fixed nondeformed Erythrocyte-DBCO (fixed CD20 EryPatch). FRET occurs when fluorophores are in close proximity, leading to a distance-dependent decrease in donor fluorescence intensity and lifetime. Thus, in this study, the FRET efficiency, calculated from the Cy3 donor lifetime in the presence and absence of the Cy5 acceptor, directly reflects the extent of CD20 clustering. Treatment with CD20 mAb produced a ring-pattern of Cy3 and Cy5 on the B cell surface. However, the resulting FRET efficiency was only marginally above baseline, indicative of minimal energy transfer, suggesting individual mAbs binding to CD20 receptors without promoting their cross-linking. Similarly, CD20 NP treatment did not induce observable spatial redistribution of CD20 on the B cell surface. The concomitant modest increase in FRET efficiency is consistent with CD20 NP–induced formation of small nanoclusters that are dispersed across the membrane. In contrast to CD20 mAb and CD20 NP, which induced only minimal CD20 clustering, CD20 EryPatch substantially promoted receptor cross-linking–induced FRET efficiency in two distinct phases (Fig. 3A). Fluorescence imaging showed that CD20 EryPatch aggregated the uniformly dispersed fluorescence into localized supramolecular condensates, accompanied by a marked rise in FRET efficiency within 3 to 6 hours. This first wave of clustering (0 to 6 hours) is likely attributable to the micrometer-scale size of CD20 EryPatch, which enables extensive surface coverage and gathers receptors across a large area. In addition, we ascribed the continued increase in cross-linking during the second wave (6 to 24 hours) to the deformability of CD20 EryPatch. To test this, we compared deformable CD20 EryPatch with fixed (nondeformable) EryPatch. Initially, fixed CD20 EryPatch produced a comparable FRET efficiency increase at 3 and 6 hours due to a similarly micrometer-scale size but plateaued thereafter, failing to show the further increase seen with deformable EryPatch at the second stage of 12 and 24 hours. In addition, CD20 EryPatch generated a more pronounced increase in membrane tension than fixed CD20 EryPatch (Fig. 3B), indicating that greater mechanical force was exerted on target B cells and their CD20 receptors. These findings suggest a two-step mechanism for CD20 EryPatch–escalated receptor cross-linking: an initial phase of widespread receptor gathering driven by coverage size, followed by a secondary phase of enhanced receptor clustering driven by deformability-dependent traction.

Given the established role of CD20 as a calcium-permeable channel, we next assessed calcium influx. In agreement with previous reports that CD20 cross-linking triggers calcium influx (*20*), CD20 EryPatch owing to its superior cross-linking efficiency elicited a stronger calcium influx than CD20 mAb, CD20 NP, or fixed CD20 EryPatch (Fig. 3C). This demonstrates that surface receptor cross-linking by CD20 EryPatch effectively translates extracellular actuation into intracellular signaling modulation. Because mitochondrial-mediated apoptosis is a canonical downstream pathway of CD20 clustering (*20*), we evaluated apoptotic response. CD20 EryPatch indeed induced the highest level of apoptosis among all groups (Fig. 3D), which was attributable to amplified manipulation of downstream cascade events, including an elevated Bax/Bcl-2 ratio (Fig. 3E), depolarization of mitochondrial membrane potential (Fig. 3, F and G), increased cytochrome c release (Fig. 3H), and subsequent caspase-3 activation (Fig. 3I).

Beyond apoptosis induction through amplified CD20 cross-linking, the biosignal changes associated with erythrophagocytosis during EryPatch transformation present a potential additional mechanism to leverage for enhanced B cell depletion. To test this hypothesis, we mixed anti-CD20 mAb-N_3_–pretargeted Raji-GFP cells with Erythrocyte-DBCO and subsequently stained for the “don’t eat me” signal CD47. Flow cytometry analysis at 0 hours revealed two distinct populations: Raji-GFP cells and erythrocytes exhibiting high CD47 expression. By 3 hours, these populations converged into a single Raji-EryPatch cluster, demonstrating successful anchoring of Erythrocyte-DBCO onto the anti-CD20 mAb-N_3_–pretargeted Raji-GFP cells via efficient bioorthogonal conjugation. Following 24 hours of incubation, a marked reduction in CD47 signal was observed within the Raji-EryPatch clusters (Fig. 3J and fig. S17). This attenuation of the don’t eat me signal corresponded with a substantial increase in the phagocytosis of the Raji-EryPatch clusters by bone marrow–derived macrophages (BMDMs) (Fig. 3K and fig. S18). In the control group, where 1,1′-Dioctadecyl-3,3,3′,3′-tetramethylindodicarbocyanine (DiD)-labeled BMDMs (BMDM-DiD) were added to the premixture of Raji-GFP cells and erythrocytes, flow cytometry initially showed three distinct populations representing Raji B cells, erythrocytes, and BMDMs. After a 4-hour coincubation, most Raji-GFP cells remained unphagocytosed. In stark contrast, when Raji-GFP were pretreated with CD20 EryPatch for 24 hours before addition of BMDM-DiD, flow cytometry initially identified only two populations: the Raji-EryPatch clusters and the BMDMs. Within 4 hours, Raji-GFP cells were predominately phagocytosed. These results confirm that the adhesion of CD20 EryPatch can act as eat me tag, attracting phagocytic BMDMs to the targeted B cells.

Together, our results demonstrate that CD20 EryPatch enhances B cell depletion via two-pronged mechanisms: inducing apoptosis through large-scale CD20 cross-linking and tagging B cells for erythrophagocytosis (Fig. 3L).

### Enhanced efficacy in NHL model

**Fig. 4.**
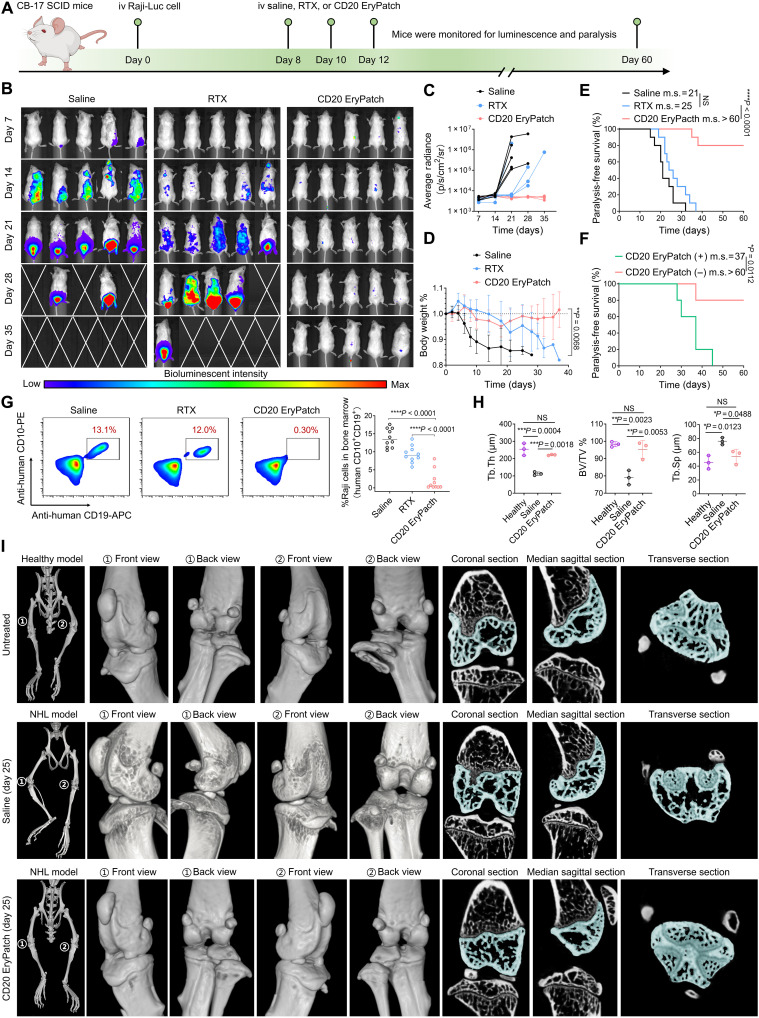
Eradication of cancerous B cell in NHL model. (**A**) Immunodeficient male CB-17 SCID mice were intravenously injected with luciferase-expressing Raji cells (Raji-Luc) on day 0 to establish NHL models. On days 8, 10, and 12, randomly divided groups received treatments with saline, anti-human CD20 mAb RTX, or CD20 EryPatch. Bioluminescent imaging was performed weekly. Survival was monitored with the onset of hindlimb paralysis as the endpoint. Created in BioRender. Mahaha, M. (2026); https://BioRender.com/hsk07r0. iv, intravenously. (**B** to **E**) Bioluminescent images (B), bioluminescent intensity (C), body weight change (D), and paralysis-free survival (E) of NHL models after treatment with saline, RTX, or CD20 EryPatch (*n* = 5 to 10). (**F**) Paralysis-free survival of the NHL mouse models receiving CD20 EryPatch with (+) or without (−) concurrent macrophage depletion (*n* = 5). m.s., median survival time. (**G**) Flow cytometry analysis of metastatic Raji cells (human CD10^+^ CD19^+^) in bone marrow (BM) of NHL models after treatment with saline, RTX, and CD20 EryPatch (*n* = 10). Analyses were performed at the experimental endpoints (onset of hindlimb paralysis or day 60, whichever came first). (**H** and **I**) Representative microcomputed tomography (micro-CT) images of hindlimb bone destruction as a result of lymphoma metastasis (H) and bone histomorphometries including bone volume/total volume (BV/TV), trabecular thickness (Tb.Th), and trabecular spacing (Tb.Sp) for knee joints (I) in NHL model receiving CD20 EryPatch on day 25. Analyses of the negative control (healthy CB-17 mouse model) and positive control (NHL model receiving saline; day 25) are shown for comparison (*n* = 3). Data are presented as mean ± SD. Statistics for survival curves in (E) and (F) are calculated by log-rank test. Statistics of others are calculated via one-way ANOVA, followed by Tukey’s multiple comparisons. NS (not significant), *P < 0.05, **P < 0.01, ***P < 0.001, and ****P < 0.0001.

NHL, the most common hematological malignancy worldwide, is predominantly of B cell origin and characterized by CD20 overexpression (*21*). Although the standard of care combines anti-CD20 immunotherapy [rituximab (RTX)] with chemotherapy, it yields complete remission in fewer than 10% of patients, and relapsed or refractory disease remains a major clinical challenge (*22*). Encouraged by in vitro results, we next evaluated CD20 EryPatch in an NHL model established by intravenous injection of human Raji B cells into immunodeficient male CB-17 severe combined immunodeficient (SCID) mice that lack functional T and B cells but retain intact macrophage activity (*23*). Mice with systemically disseminated Raji B cell lymphomas then received intravenous treatment with saline, RTX, or CD20 EryPatch on days 8, 10, and 12. Survival was monitored until the onset of hindlimb paralysis caused by bone destruction due to lymphoma metastasis (Fig. 4A). For in vivo application of CD20 EryPatch, a 5-hour interval between the intravenous injections of RTX-N_3_ and Erythrocyte-DBCO was adopted. This timing was based on established strategies for consecutive two-step therapies that use an RTX-based pretargeting module (*24*). Our pharmacokinetic data confirmed this timing, showing RTX-N_3_ reached a steady-state blood concentration at ~5 hours postinjection (fig. S19). This plateau indicated an optimal window for administering Erythrocyte-DBCO, as the blood concentration of free RTX-N_3_ (unbound to B cells) was limited, minimizing interference with the subsequent bioorthogonal reaction. When Erythrocyte-DBCO was injected at this 5-hour interval, the concentration of RTX-N_3_ declined rapidly instead of maintaining its plateau, implying enhanced clearance of B cell targets. To validate the in vivo click reaction, we used a FRET pair, conjugating Cy5 to RTX-N_3_ and Cy5.5 to Erythrocyte-DBCO. Blood samples from NHL mice treated with CD20 EryPatch (RTX-N_3_-Cy5 → Erythrocyte-DBCO-Cy5.5) showed a strong FRET signal relative to the control (RTX-N_3_-Cy5 → Erythrocyte-Cy5.5), confirming an energy transfer driven by their close proximity (fig. S20A). This result confirms that CD20 EryPatch executes bioorthogonal click reaction in vivo. We then constructed a blood-circulating memetic device, powered by a peristaltic pump to recreate murine hemodynamic environment for detailed analysis of CD20 EryPatch binding and transforming behaviors on B cells during circulation. Within this system, CD20 EryPatch bound to Raji B cells rapidly, eventually reaching a maximum of three EryPatches per B cell within 3 hours (fig. S20B). Subsequent morphological analysis revealed a time-dependent discocyte-to-echinocyte transition of the bound EryPatch on B cells, evident from 6 hours and pronounced by 12 to 24 hours (fig. S20C). Concurrently, CD47 expression within CD20 EryPatch–B cell clusters decreased progressively from 6 to 24 hours (fig. S20D). These results demonstrate that CD20 EryPatch remodeling is synchronized with progressive CD47 loss, both occurring after binding to B cells. Furthermore, as shown in fig. S21, the pharmacokinetics and biodistribution of intravenously injected Erythrocyte-DBCO were substantially altered compared to unmodified erythrocytes. The blood concentration profiles of Erythrocyte-DBCO and unmodified erythrocytes were similar within the first 3 hours postinjection. However, consistent with the onset of morphological remodeling and time-dependent CD47 loss observed after 6 hours of circulation (fig. S20D), the blood concentration of Erythrocyte-DBCO declined sharply starting at 4 hours and was nearly cleared by 24 hours, yielding a terminal half-life of 11.5 hours (fig. S21A). In contrast, native murine erythrocytes, which typically circulate for weeks (half-life >17 days), remained stable in blood over the same period. Notably, we detected a pronounced accumulation of Erythrocyte-DBCO in the spleen 24 hours postinjection (fig. S21B), which makes sense because senescent or damaged erythrocytes are predominantly cleared by macrophages in spleen (*25*). The senescence-like features of Erythrocyte-DBCO, characterized by deformation and CD47 loss, appear to initiate erythrophagocytosis, leading to its accelerated blood clearance and ultimate removal by the spleen. Collectively, these findings demonstrate that Erythrocyte-DBCO can induce erythrophagocytosis, a key mechanism underlying CD20 EryPatch–mediated B cell depletion.

In an imageable NHL model using luciferase-expressing Raji cells (Raji-Luc; Fig. 4, B and C), the control mice (saline) exhibited rapid disease progression after 14 days, with bioluminescence revealing abundant tumor occupation of B cell lymphoma dissemination hotspots (tibiae, femora, and spine) by day 21, ultimately resulting in hindlimb paralysis in three-fifth mice by day 28 and severe tumor burden in the remainder, followed by 100% mortality by day 35. While the clinically used anti-CD20 mAb (RTX) moderately inhibited tumor growth and delayed whole-body bioluminescence progression to a limited extent, most RTX-treated mice still showed extensive lymphoma dissemination by day 28 and succumbed by day 35. Notably, CD20 EryPatch demonstrated substantial therapeutic improvement over RTX, achieving complete tumor elimination in all treated mice, with a luciferase signal reduced to background levels during treatment. In a separate batch of the same in vivo experiment, body weight changes (Fig. 4D) and survival outcomes (Fig. 4E) correlated with the bioluminescence data. Saline-treated mice rapidly developed hindlimb paralysis, exhibiting a median survival of 21 days and 0% survival at the endpoint. RTX yielded a modest extension in median survival to 25 days but ultimately resulting in complete mortality accompanied by considerable weight loss due to disease progression. In contrast, CD20 EryPatch substantially prolonged animal survival and demonstrated substantially superior anticancer efficacy, with 80% of mice remaining paralysis free and maintaining stable body weight for over 60 days. In a subsequent study, NHL mice were subjected to macrophage ablation before CD20 EryPatch therapy (Fig. 4F). Concurrent macrophage depletion partially abolished the therapeutic benefit of CD20 EryPatch with the median survival reduced from over 60 days to 37 days but still outperforming RTX that had a median survival of only 25 days. These results provide supporting evidence that the antilymphoma efficacy of CD20 EryPatch is a combined result of apoptosis enhancement and erythrophagocytosis involvement.

At the therapy endpoint (defined by the onset of hindlimb paralysis or day 60, whichever occurred first), we quantitatively analyzed the dissemination of lymphoma Raji B cells (human CD10^+^CD19^+^) in the femoral bone marrow (BM) using flow cytometry (fig. S22). Substantial proportions (~10 to 17%) of Raji cells were detected in the BM of paralyzed mice across all treatment groups, including all saline- and RTX-treated mice, as well as two paralyzed mice from the CD20 EryPatch group. In contrast, the other 8 of 10 CD20 EryPatch–treated mice, which remained paralysis-free long-term survivors, exhibited minimal Raji cell infiltration (average <1%) in the BM (Fig. 4G). We also performed three-dimensional microcomputed tomography (3D micro-CT) imaging on the hindlimbs, the known hotspots for B cell NHL metastasis, in Raji lymphoma–bearing mice receiving CD20 EryPatch treatment. The results revealed extensive bone heterogeneity in NHL controls, characterized by severe trabecular loss and architectural disruption, particularly in the proximal tibia near the knee joint. These changes indicate abnormal osteoclast activation and bone remodeling stimulated by lymphoma metastases. In contrast, mice treated with CD20 EryPatch showed no signs of bone pathology, with preservation of bone architecture comparable to healthy controls (Fig. 4H). Quantitative analysis of bone volume/total volume (BV/TV), trabecular thickness (Tb.Th), and trabecular spacing (Tb.Sp) confirmed that CD20 EryPatch maintained bone microstructural parameters at levels similar to healthy controls (Fig. 4I).

### Enhanced efficacy in SLE model

**Fig. 5.**
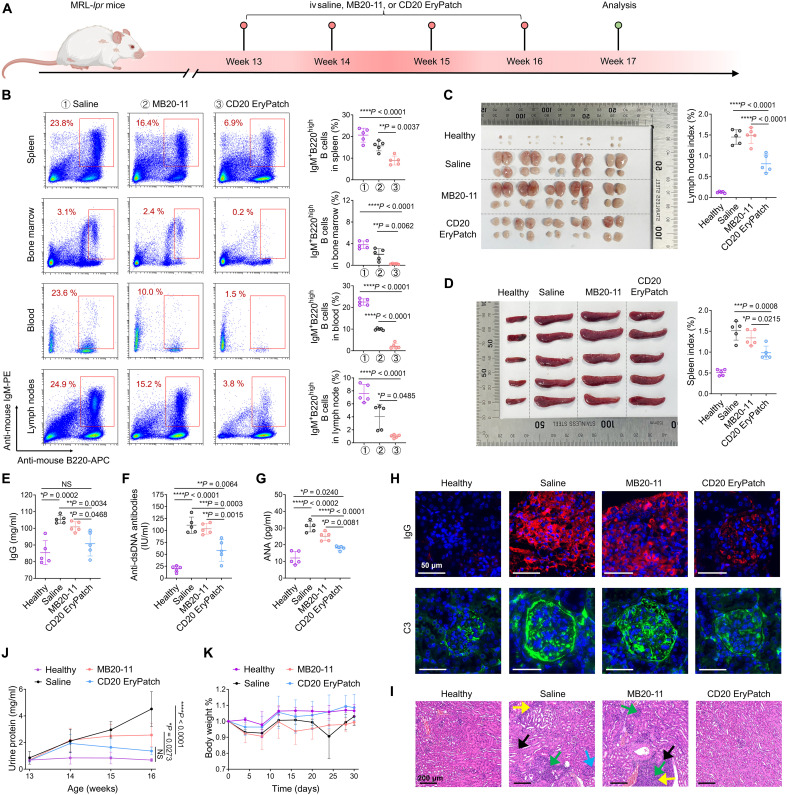
Alleviation of disease symptoms in SLE model. (**A**) Genetically defective MRL-*lpr* mice, which carry a Fas gene mutation impairing lymphocyte apoptosis and spontaneously develop human SLE-like disease, start receiving intravenous treatments at 13 weeks of age, administered in four weekly cycles of saline, anti-mouse CD20 mAb (MB20-11), or CD20 EryPatch (MB20-11–N_3_ → Erythrocyte-DBCO). Age-matched C57BL/6J mice without recessive *lpr* gene mutation served as healthy control. Mice were euthanized at week 17 for endpoint analyses. Created in BioRender. Mahaha, M. (2026); https://BioRender.com/hsk07r0. (**B**) Flow cytometry scatter plot representation and quantitative analysis of mature IgM^+^B220^high^ B cell depletion in the spleen, BM, peripheral blood, and lymph nodes at the endpoint. (**C** and **D**) The extent of lymphadenopathy and splenomegaly, shown by representative images and organ weight indices for lymph nodes (C) and spleens (D) from mice treated with saline, MB20-11, or CD20 EryPatch. (**E** to **G**) Levels of autoantibody production including total IgG (E), anti–double-stranded DNA (dsDNA) (F), and antinuclear antibodies (ANA) (G) measured in serum at the treatment endpoint. (**H**) Representative histological images of deposits in kidney sections stained with immunofluorescence for IgG (red) and complement C3 (green). Scale bars, 50 μm. (**I**) Kidney sections stained with hematoxylin and eosin (H&E); arrows indicate key pathologies: tubular atrophy (yellow), inflammatory infiltration (green), tubular edema (black), and crescent formation (blue). Scale bars, 200 μm. (**J** and **K**) Urine protein level (J) and body weight change (K) in mice throughout the treatment period. *n* = 5 animals per group. Data are presented as mean ± SD. Statistics for proteinuria urine (J) is calculated by two-way ANOVA. Statistics of others are calculated via one-way ANOVA, followed by Tukey’s multiple comparisons. NS (not significant), *P < 0.05, **P < 0.01, ***P < 0.001, and ****P < 0.0001.

B cells play a central role in SLE by producing pathogenic autoantibodies that form immune complexes, which deposit in tissues and activate complement, ultimately mediating inflammation and tissue damage; in addition, through antigen presentation and cytokine production, they promote autoreactive T cell activation, thereby sustaining a self-perpetuating cycle of autoimmune injury (*16*, *26*). RTX, originally developed for eliminating B cell malignancies, was the first BCDT used off-label in patients with SLE (*17*). Although it failed to meet primary endpoints in randomized controlled trials for SLE, likely due to inadequate suppression of autoreactive B cell survival despite effective binding, it has laid the groundwork for several B cell targeting modalities for treating SLE, including enhanced mAbs, chimeric antigen receptor T cells (CAR T cells), or bispecific T cell engagers (*16*). Given the enhanced B cell depletion capability of CD20 EryPatch, we assessed its efficacy in genetically defective MRL-*lpr* mice. This model replicates human SLE through a Fas gene mutation that prevents lymphocyte apoptosis, resulting in a spontaneous disease characterized by massive lymphadenopathy, splenomegaly, autoantibody production, and fatal immune complex–mediated glomerulonephritis (*27*). Because anti-human CD20 mAbs do not cross react with murine CD20 (*28*), anti-mouse CD20 antibody MB20-11 was selected to create CD20 EryPatch platform (MB20-11–N_3_ → Erythrocyte-DBCO, with 5-hour interval). Weekly treatment of MRL-*lpr* mice was initiated at 13 weeks of age, a time point when MRL-*lpr* mice start exhibiting spontaneous lupus-like disease (Fig. 5A). Upon endpoint analysis following four doses, CD20 EryPatch effectively depleted mature B cells [defined as immunoglobulin M (IgM)^+^B220^high^] across the spleen, BM, blood, and lymph nodes. The residual B cell frequency was significantly lower in the CD20 EryPatch group compared to both control groups receiving saline or MB20-11 treatment, demonstrating its superior capacity for systemic B cell depletion (Fig. 5B and fig. S23).

Lymphatic hyperplasia and splenomegaly, key pathological features of SLE driven by aberrant lymphocyte activation, were significantly ameliorated by CD20 EryPatch treatment. In contrast, MB20-11 did not yield a statistically significant reduction in lymph node size compared with saline-treated MRL-*lpr* control (Fig. 5C). A similar efficacy profile was observed for splenomegaly, with CD20 EryPatch showing superior mitigation compared to MB20-11 (Fig. 5D). Consistent with its enhanced B cell depletion, CD20 EryPatch prevented their secretion of key lupus-associated autoantibodies, as shown by significantly lower levels of serum IgG (Fig. 5E), anti–double-stranded DNA (dsDNA) antibodies (Fig. 5F), and antinuclear antibodies (ANA) (Fig. 5G) as compared to saline and MB20-11 treatments. Renal dysfunction from glomerulonephritis is a leading cause of mortality among SLE-afflicted individuals. This pathology results from the deposition of immune complexes containing IgG and complement factors such as C3 within the kidneys, which initiates inflammatory damage and functional impairment (*29*). Immunolabeling of the kidney sections revealed extensive IgG and C3 deposits in glomeruli of both saline- and MB20-11–treated MRL-*lpr* groups but observable reduction in CD20 EryPatch group (Fig. 5H). Kidney histopathology analysis confirmed severe renal injury in saline- and MB20-11–treated mice, characterized by tubular edema, atrophy, crescent formation, and inflammatory cell infiltration. However, these pathological features were ameliorated in CD20 EryPatch–treated mice (Fig. 5I). In addition, urine protein was measured as a reflection of renal damage. As shown in Fig. 5J, saline-treated mice exhibited a rapid increase in urinary protein excretion, consistent with accelerated disease progression. While MB20-11 treatment mildly attenuated this trend, CD20 EryPatch significantly suppressed proteinuria elevation and maintained lower levels at the endpoint, demonstrating a superior therapeutic benefit against lupus nephritis. Notably, while CD20 EryPatch did not fully reverse SLE to the normal level of healthy controls without the *lpr* mutation, it demonstrated substantial therapeutic benefits by effectively halting disease progression and markedly alleviating disease symptoms compared to saline and MB20-11 (Fig. 5, C to J). Moreover, no significant body weight loss was observed during the CD20 EryPatch treatments (Fig. 5K).

### Enhanced efficacy in RA model

**Fig. 6.**
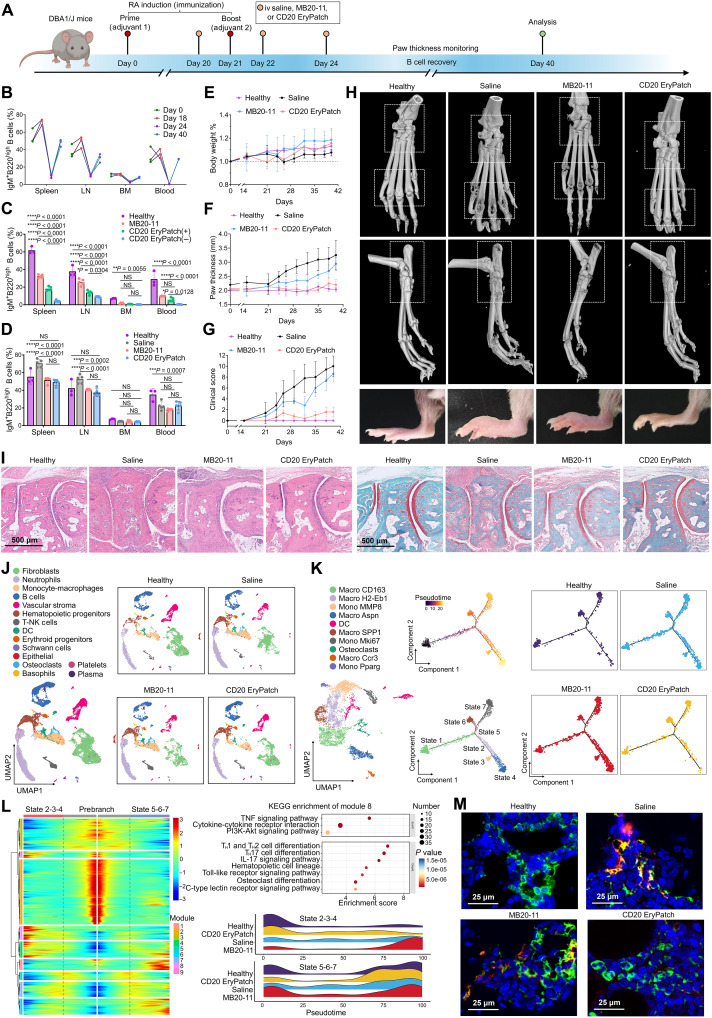
Retardation of disease progression in CIA model. (**A**) DBA/1J mice were immunized with bovine CII emulsified in complete Freund’s adjuvant (CFA) on day 0 and received a booster immunization on day 21. Treatments were given on days 20, 22, and 24. Created in BioRender. Mahaha, M. (2026); https://BioRender.com/hsk07r0. (**B**) Percentage changes in mature B cells (IgM^+^B220^high^) in spleen, lymph nodes, BM, and peripheral blood following CD20 EryPatch treatment, showing healthy (day 0), diseased (day 18), B cell depleted (day 24), and B cell recovered (day 40) states. LN, lymph nodes. (**C** and **D**) Analysis of B cell depletion across tissues on day 24 (C) and day 40 (D) after treatment with saline, MB20-11, and CD20 EryPatch with (+) or without (−) concurrent macrophage depletion (*n* = 5). (**E** to **G**) Disease monitoring in arthritic mice including body weight (E), paw thickness (F), and clinical arthritis score (G) until day 40 (*n* = 5). (**H**) Micro-CT images and photographs of hind ankle joints of normal and arthritic mice after different treatments on day 40. (**I**) Histology of ankle joint sections stained with H&E (left) and Safranin O–Fast Green (right). Scale bars, 500 μm. (**J**) Unified manifold approximation and projection (UMAP) plot visualizing 15 distinct cell clusters identified from single cells isolated from ankle joint tissues. DC, dendritic cell. (**K**) Reference UMAPs highlighting monocyte-macrophage population (left) and pseudotime trajectory analysis inferring macrophage differentiation path (right). (**L**) Pseudotime trajectory analysis of macrophages. (**M**) Immunofluorescence staining of knee joint sections for macrophages (F4/80^+^) and M1 proinflammatory macrophages (F4/80^+^ CD86^+^). Green indicates F4/80, red indicates CD86, and blue indicates cell nuclei. Scale bars, 25 μm. Data are presented as mean ± SD with statistics calculated by two-way ANOVA. NS (not significant), *P < 0.05, **P < 0.01, ***P < 0.001, and ****P < 0.0001.

To explore the potential of CD20 EryPatch in treating other autoimmune diseases, we conducted a proof-of-concept study in rheumatoid arthritis (RA), wherein B cells are one of the crucial instigators to orchestrate autoimmune response for massive recruitment and activation of macrophages that act as the primary executors of inflammation and joint destruction (*30*). DBA/1J mice immunized with type II collagen (CII) on day 0 and boosted on day 21 to induce collagen-induced arthritis (CIA) (*31*) received three doses of CD20 EryPatch (MB20-11–N_3_ → Erythrocyte-DBCO) on days 20, 22, and 24 (Fig. 6A). Dynamic changes in mature B cell percentages were analyzed across spleen, lymph nodes, BM, and peripheral blood at four time points: day 0 (healthy state, preimmunization), day 18 (diseased state, post–CIA induction), day 24 (immediately after final treatment), and day 40 (16 days posttreatment). CD20 EryPatch treatment produced highly comparable B cell depletion-to-recovery curves across all tissues analyzed, characterized by a slight increase in B cell percentage on day 18 coinciding with RA onset, a sharp decline by day 24 suggesting effective depletion, and a rebound by day 40 reflecting eventual B cell repopulation after treatment cessation (Fig. 6B and fig. S23).

For comparative analysis of B cell depletion, assessments were performed on day 24 in CIA mice treated with saline, MB20-11, or CD20 EryPatch. Regardless of concurrent macrophage depletion, CD20 EryPatch induced stronger B cell depletion than MB20-11 in the spleen, lymph nodes, and blood although not in the BM. However, macrophage ablation attenuated the B cell depleting effect of CD20 EryPatch when compared to treatment without macrophage depletion (Fig. 6C and fig. S23). These results support the hypothesis that, in addition to enhanced apoptosis, erythrophagocytosis contributes to B cell depletion by CD20 EryPatch during RA treatment. Furthermore, mice experienced B cell recovery after CD20 EryPatch treatment ended, with proportions returning to near-normal levels in most tissues by day 40, comparable to those in healthy controls (Fig. 6D and fig. S23). This recovery is likely because CD20 is specifically overexpressed during intermediate stages of B cell development (from pre-B to mature B cells) but is absent on early stages of hematopoietic stem cells (HSCs). Consequently, similar to other CD20-targeting therapies, CD20 EryPatch depletes mature B cells while preserving the potential for de novo B cell regeneration from HSCs (*28*, *32*).

Mice were also monitored for body weight and arthritis development. All groups showed a steady increase in body weight, indicating no treatment-related toxicity (Fig. 6E). Paw thickness increased rapidly in saline-treated CIA mice following the second immunization. MB20-11 delayed paw swelling but ultimately failed to halt disease progression. Conversely, CD20 EryPatch treatment effectively flattened the growth curve, resulting in paw thickness comparable to that of healthy DBA/1J mice and significantly lower than that in the saline and MB20-11 groups (Fig. 6F). Clinical arthritis scores, which assess joint inflammation and structural damage based on redness and swelling, showed a similar trend (Fig. 6G). At the endpoint, ankle joints were harvested and evaluated by micro-CT. Severe bone loss and deformity were observed in the digit joints of saline-treated CIA mice. Their ankle joints also exhibited substantial bone and cartilage erosion, with irregular bone architecture. While MB20-11 treatment modestly mitigated these pathological features, the CD20 EryPatch group exhibited smooth bone surfaces and relatively intact joint structures that closely resembled those of healthy mice (Fig. 6H). Consistent with this, CD20 EryPatch treatment resulted in markedly reduced histopathological inflammation and ankle joint swelling, with joint morphology similar to healthy controls (Fig. 6H and fig. S24). Histological analyses [hematoxylin and eosin (H&E), Safranin O–Fast Green, and Masson’s trichrome staining] of ankle joints (Fig. 6I and fig. S25) and knee joints (fig. S26) revealed severe arthritic pathology in CIA mice including synovial hyperplasia, inflammation, cartilage erosion, and collagen deposition, which were only marginally improved by MB20-11. Conversely, CD20 EryPatch treatment markedly attenuated these changes, with intact cartilage, minimal inflammation, reduced synovial hyperplasia, and no detectable pannus.

We further conducted single-cell RNA sequencing (scRNA-seq) on synovial tissue from healthy, saline-, MB20-11–, and CD20 EryPatch–treated mice at day 40 to establish a comprehensive transcriptional atlas. Following quality control and batch-effect correction, 32,506 high-quality cells were clustered into 15 major cell populations (Fig. 6J), defined by canonical markers (fig. S27). Figure 6J showed the unified manifold approximation and projection (UMAP) visualization and the distribution of cell clusters across different treatment groups, which includes monocyte-macrophages, B cells, natural killer T (T-NK) cells, etc. Figure S28A revealed significant gene expression changes. Notably, Gene Ontology (GO), Kyoto Encyclopedia of Genes and Genomes (KEGG), and gene set enrichment analyses revealed an overactivated trend of RA-related signaling pathways in CIA mice treated with both saline and MB20-11, including inflammatory response and chemokine signaling pathways. In contrast, CD20 EryPatch treatment effectively down-regulated these pathways, indicating a gradual restoration of physiological states to those observed in healthy mice (fig. S28, B to D). To further identify the pathways modulated by CD20 EryPatch, we intersected the mRNAs up-regulated in the saline group and down-regulated in the CD20 EryPatch group, identifying 337 differentially expressed mRNAs for functional enrichment analysis (fig. S29). The KEGG and GO analyses indicated that CD20 EryPatch treatment significantly inhibited several vital signaling pathways, including cytokine-cytokine receptor interaction, tumor necrosis factor (TNF), nuclear factor κB, and interleukin-17 (IL-17) signaling pathways (highlighted in red) while modulating immune and inflammatory response processes (highlighted in green) (fig. S29, B and C). To validate these findings, we quantified serum levels of autoantibody (IgG; fig. S30A), proinflammatory cytokines [transforming growth factor–β1 (TGF-β1), TNF-α, IL-17, and IL-1β; fig. S30A], and anti-inflammatory IL-10 (fig. S30B) by enzyme-linked immunosorbent assay (ELISA). CD20 EryPatch treatment significantly reduced autoantibody and proinflammatory cytokine levels while elevating anti-inflammatory cytokines. B cells promote synovial joint destruction by secreting autoantibodies and cytokines that recruit macrophages, the direct cellular mediators of inflammation and damage. Motivated by our earlier data revealing differences in monocyte-macrophage numbers and IL-17 signaling (a key inducer of proinflammatory macrophage polarization), we therefore focused our investigation on monocyte-macrophage subpopulations. We defined 10 transcriptionally distinct subtypes and subsequently performed pseudotime analysis on macrophages (Fig. 6K and fig. S31), and two distinct progression routes were revealed along the pseudotime paths from state 1 to state 2-3-4/5-6-7. In particular, cells from saline and MB20-11 groups were significantly enriched in state 2-3-4, which were rarely observed in the healthy and CD20 EryPatch groups. To clarify this phenomenon, we conducted a dynamic gene expression analysis on variable genes within the pseudotime trajectory (Fig. 6L, left). Among the nine modules, the gene expression levels of modules 8 and 9 were high in state 2-3-4 while low in state 5-6-7. KEGG analysis revealed an enrichment in immune- and inflammatory-related signaling pathways within these modules (Fig. 6L, top right, and fig. S32). Quantitative analysis of ridge plots revealed distinct cellular density distributions across groups (Fig. 6L, bottom right). The saline and MB20-11 groups showed higher enrichment in state 2-3-4, indicating abundant infiltration of proinflammatory macrophages in murine synovial tissues. In contrast, the CD20 EryPatch group exhibited a density pattern in state 2-3-4 attenuated and comparable to healthy mice, demonstrating its efficacy in reducing proinflammatory macrophage infiltration. Consistent with this, a fewer number of proinflammatory F4/80^+^CD86^+^ M1 macrophages were observed in the arthrosis of CIA mice treated with CD20 EryPatch (Fig. 6M and fig. S33). Together, these results demonstrate that the enhanced B cell depletion capability of CD20 EryPatch translates to superior efficacy in treating RA.

A comprehensive biosafety assessment of CD20 EryPatch was then conducted in healthy, immunocompetent DBA/1J mice following three administered doses. Complete blood counts covering leukocytes, erythrocytes, and platelets showed no evidence of anemia after the treatment, with all hematological parameters remaining within normal ranges and comparable to untreated controls (fig. S34A). Furthermore, no splenomegaly, indicative of splenic sequestration, was observed in CD20 EryPatch–treated mice (fig. S34B). Although EryPatch-mediated B cell depletion involves erythrophagocytosis that accelerates blood clearance (fig. S21A) and leads to splenic removal (fig. S21B), the dose of Erythrocyte-DBCO used (1 × 10^7^ cells) represents <1% of total circulating erythrocytes in a 20-g mouse. This low, hemodynamically well-tolerated dose, consistent with established erythrocyte-based platforms (*25*, *33*, *34*), explains the absence of induced splenomegaly. In addition, assessment of immunogenicity revealed no aberrant activation of T cells (fig. S34C) or macrophages (fig. S34D) in lymphoid organs (spleen and lymph nodes) of CD20 EryPatch–treated mice, while serum levels of key proinflammatory (TNF-α, IL-6, IL-17, and IL-1β) or regulatory (IL-4 and TGF-β) cytokines remained unchanged compared to untreated controls (fig. S34E). Similarly, serum chemistry analysis detected no signs of hepatotoxicity or renal impairment (fig. S34F), and histopathological examination of major organs showed no pathological abnormalities (fig. S34G). Collectively, these results demonstrated that CD20 EryPatch did not alter hematological parameters, elicit immune or inflammatory response, or cause organ damage.

Having demonstrated the efficient B cell depletion by CD20 EryPatch and subsequent B cell recovery 16 days after the treatment cessation (Fig. 6B), we further evaluate whether the repopulated B cell retained normal function and could mount an immune response to immunization. DBA1/J mice received three doses of CD20 EryPatch and were allowed 16 days for B cell recovery. Immunization was performed via subcutaneous injection of ovalbumin (OVA) emulsified in alum on day 1 and boosted on day 8, and B cell subsets were analyzed before immunization (day 0) and after the boost (day 15) (fig. S35A). Before immunization (day 0), most B cells in the BM, the site where B cells originate, displayed an immature phenotype (IgD^−^CD27^−^IgG^−^). Meanwhile, the repopulated B cells posttreatment were predominantly naïve (IgD^+^CD27^−^) in blood, spleen, and lymph nodes (fig. S35B). This is suggestive of an immune reset, similar to what has been reported in the use of lipid nanoparticles to generate anti-CD19 CAR T cells in vivo, leading to deep B cell depletion in cynomolgus monkeys (*32*). Following immunization, the frequencies of memory B cells including unswitched (IgD^+^CD27^+^) and switched (IgD^−^CD27^+^) memory B cells increased significantly in the tissues by day 15. Consistent with this, serum antibody titers of OVA-specific IgG, IgG1, and IgG2a increased significantly after immunization (fig. S35C). These results indicate that B cell repopulation after CD20 EryPatch therapy preserves a functional immune repertoire.

## DISCUSSION

Cell surface receptor cross-linking occurs when a multivalent ligand tethers neighboring receptors together, physically clustering them into aggregates. This forced proximity generates outside-in mechanotransduction, which powerfully converts the external binding event into a diverse and amplified instructional signal inside the cell. This process is critical for activating receptors that, in their monomeric state, are functionally inactive and incapable of initiating downstream signaling (*1*, *2*). Artificial cross-linking strategies using a diverse array of synthetic platforms, such as multivalent liposomes (*35*), nanoparticles (*36*), adhesive micropatches (*18*, *37*), self-assembling polymers (*13*, *14*), and retractable DNA nanostrings (*9*, *38*), are being used to cluster specific receptors for therapeutic benefits, including inducing apoptosis via death receptors (*10*), inhibiting metastasis via CXCR4 (*13*), activating immune response via T cell receptors (*38*), and treating autoimmune disease via CD95 (*39*). In this study, we developed CD20 EryPatch for enhanced B cell depletion. This platform differs from existing cross-linking approaches by leveraging three dynamic features of lipid-inserted erythrocyte: (i) its initial large surface area to enable extensive CD20 coverage on B cells; (ii) its discocyte-to-echinocyte transition to drive large-scale CD20 cross-linking, leading to massive downstream apoptosis in B cells; and (iii) a biological alteration of CD47 loss to transform EryPatch into an eat me tag on B cells for further clearance via erythrophagocytosis.

First, the spatial scale of receptor clustering critically determines the strength of downstream signaling. For instance, NK cells are more potently activated when NK receptor targeting antibodies are presented on microparticles (4 to 8 μm) rather than nanoparticles (~200 nm) because the microparticle diameter matches the length scale of receptor clusters (3 to 6 μm) typically formed on NK cells during an immune synapse (*18*). Similarly, we demonstrated that compared to CD20 NP, which only form multiple small, dispersed clusters (Figs. 2D and 3A), CD20 EryPatch that governs localized, larger-scale clustering covering extensive B cell surface areas (Figs. 2, B to F, and 3A) is more effective at transducing signals and inducing apoptosis (Fig. 3, B to I). Using erythrocytes as a scalable and biocompatible module, CD20 EryPatch overcomes the size constraints of conventional synthetic materials and enables micrometer-scale receptor cross-linking across broad regions of the B cell surface.

Second, upon binding to B cells, the CD20 EryPatch does not remain statically attached to the cell surface; rather, it undergoes a dynamic discocyte-to-echinocyte transition (Fig. 2B). This morphological shift is triggered by membrane stress resulting from altered fluidity and surface tension following lipid insertion via DSPE–PEG-2000 (Fig. 1G and figs. S6 to S9). Mature erythrocytes, which lack major organelles and have a high surface area–to–volume ratio along with ease of modification, represent an ideal biomimetic delivery vehicle (*40*). However, without appropriate drug loading technology, their susceptibility to hypotonic drug encapsulation intracellularly or to membrane-anchored conjugation via lipid insertion often compromises natural longevity in circulation, thereby undermining their potential as long-circulating carriers. In this study, we repurpose this liability as a strategic opportunity: By leveraging the driving forces generated from dynamic biointerface remodeling, we transform this former foe into a functional mechanism that enhances receptor cross-linking. Within the first 6 hours, before the deformation of CD20 EryPatch occurs (Fig. 2B), both geometry-fixed and deformable CD20 EryPatches cross-link CD20 into localized clusters, with significantly higher efficiency than CD20 NPs (Fig. 3A), owing to their micrometer-scale size coverage that gathers receptor over a large cell surface area. After 6 hours, however, as the discoidal EryPatch begins transitioning into an echinocyte (Fig. 2B), their behavior diverges: While CD20 cross-linking extent plateaus in fixed EryPatch, it continues to increase in deformable EryPatch (Fig. 3A), leading to stronger apoptosis induction by the latter (Fig. 3D). These results support a two-step receptor cross-linking mechanism by CD20 EryPatch: initial size-driven receptor aggregation followed by deformability-driven clustering enhancement.

Third, a further advantage of CD20 EryPatch is its ability to emit phagocytic signal for B cell clearance. Lipid insertion into the erythrocyte membrane induces stress that progressively reduces CD47, a surface protein that signals macrophages to avoid engulfment (Fig. 1E). This change effectively converts the CD20 EryPatch into an eat me tag attached to B cells, facilitating their recognition and phagocytosis by macrophages (Fig. 3K). The critical role of this pathway was also confirmed in macrophage-ablated mice, where the loss of phagocytic activity shortened survival in a B cell malignancy model (Fig. 4F) and impaired B cell depletion in an autoimmune model (Fig. 6C), demonstrating that erythrophagocytosis contributes to the therapeutic efficacy of CD20 EryPatch.

B cell dysregulation can lead to malignant proliferation, excessive autoantibody production, and aberrant proinflammatory cytokine secretion, which is a root cause of various B cell–related diseases, including B cell lymphoma and autoimmune disorders (*16*, *17*, *26*, *29*, *30*). CD20 is specifically expressed on pre-B cells and mature B cells with increasing abundance during differentiation. It is absent on HSCs, plasma cells, and other normal tissues, making it an ideal target for depleting pathogenic B cells while preserving the capacity for healthy B cell reconstitution and plasma cell–mediated immune surveillance (*16*, *17*). In this study, we demonstrated that BCDT using CD20 EryPatch effectively eradicated malignant B cells in NHL model (Fig. 4), alleviated systemic inflammation in SLE model (Fig. 5), and delayed joint inflammation and destruction in RA model (Fig. 6), with therapeutic efficacy better than CD20 mAb. Furthermore, the treatment was well tolerated, with no notable toxicity observed during or after the treatment (fig. S34). Recent studies indicate that CD20 is also expressed at low levels on a rare subset of T cells. In patients with multiple sclerosis, these CD3^+^CD20^low^ T cells correlate with disease severity and produce elevated levels of proinflammatory cytokines, and they can be efficiently depleted by RTX. Thus, anti-CD20 therapy may be effective, in part, through removal of CD20^+^ T cells (*17*). However, controversy remains regarding whether CD20^+^ T cells are reactive in immunosurveillance, directly pathogenic, or both (*41*). Therefore, whether the efficacy of CD20 EryPatch involves depletion of CD20^+^ non-B cells, and whether inevitable CD20 cross-linking on non-B cells leads to off-target toxicity, warrants specific attention in future studies.

From a practical standpoint, the two-step regimen for CD20 EryPatch offers the advantage of pretargeting that functionally separates B cell targeting by CD20 mAb-N_3_ from subsequent receptor cross-linking by deformable Erythrocyte-DBCO. This creates a modular and generalizable platform. The pretargeting module (mAb-N_3_) can be considered an “off-the-shelf” reagent that could, in principle, be swapped for antibodies against other cell surface targets for different diseases, while the cross-linking module (Erythrocyte-DBCO) remains unchanged, enabling an enhanced flexibility for a broad therapeutic application. In addition, both modules are simple to fabricate and administer independently. CD20 mAb-N_3_ is a stable, storable reagent, while Erythrocyte-DBCO is prepared via a short incubation to insert DBCO-PEG-DSPE into erythrocytes immediately before use. In contrast, preassembly before injection would require an additional bioorthogonal reaction step to conjugate the antibody to the erythrocyte. This reaction would necessitate precise optimization: The incubation time must be long enough to ensure efficient coupling yet short enough to prevent the premature deformation of lipid-inserted erythrocytes. This complexity could compromise scalability, reproducibility, and the functional integrity of the final product. For translation implication, a personalized approach using patient-specific autologous erythrocytes would be required to avoid potential immunogenicity concerns. Despite known differences in size, membrane structure, and surface area between mouse and human erythrocytes, their susceptibility to membrane perturbation by lipid insertion of DBCO–PEG-2000–DSPE is likely conserved. However, sensitivity may vary, leading to differences in the rates or extents of Erythrocyte-DBCO deformation and CD47 down-regulation, which requires further dosage optimization to replicate comparable effects in human erythrocytes.

In summary, we developed CD20 EryPatch as a proof-of-concept platform for enhanced BCDT. The morphological and biological changes in CD20 EryPatch facilitate B cell apoptosis via large-scale CD20 cross-linking and B cell eat me tagging for erythrophagocytosis, respectively. This strategy demonstrates superior efficacy over standard anti-CD20 antibody therapy against B cell disorders, including NHL, SLE, and RA.

## MATERIALS AND METHODS

### Materials

RTX (Rituxan) was purchased from Roche Pharma AG (Basel, Switzerland). MB20-11 (InVivoMAb anti-mouse CD20) was purchased from BioXcell (New Hampshire, USA). Tris(2-carboxyethyl) phosphine (TCEP) was purchased from Share-bio (Shanghai, China). N_3_–PEG-1000–maleimide, DBCO–PEG-2000–DSPE, and sulfo-Cy3 *N*-hydroxysuccinimide (NHS) ester were purchased from Tanshtech (China). Sulfo-Cy5 NHS ester was bought from Yusi Pharmaceutical Technology Co. Ltd. (Chongqing, China). DBCO-Cy5 was bought from Aladdin Biotech Co. Ltd. (Shanghai, China). Alsever’s solution, Biotin Quantitative Assay Kit, and Hoechst were provided by Solarbio Science & Technology Co. Ltd. (Beijing, China). Cholesterol-PEG-Biotin was purchased from Macklin (Shanghai, China). FITC–PEG-2000–DSPE was provided by Ruixi Biotechnology Co. Ltd. (Xian, China). Flipper-TR was bought from Spirochrome (Switzerland). DiD fluorescent dye and granulocyte-macrophage colony-stimulating factor protein (mouse) were purchased from MedChemExpress (USA). Sialic acid assay kit (A036-1-1) and urine protein test kit (C035-2-1) were purchased from Nanjing Jiancheng Bioengineering Institute (Nanjing, China). CellTrace Far Red was provided by Thermo Fisher Scientific Co. Ltd. (Massachusetts, USA). Fluo-4 AM calcium assay kit (S1061M), Bax mAb (AB026), anti–cytochrome c mAb (AC908), caspase-3 activity and mitochondrial membrane potential detection kit for live cell (C1073S), and ACK lysis buffer (C3702) were provided by Beyotime Biotechnology (Shanghai, China). Annexin V–FITC/propidium iodide (PI) apoptosis detection kit and clodronate liposomes were provided by Yeasen Biotechnology Co. Ltd. (Shanghai, China). Bcl-2 mAb (HA723111), iFluor 647–conjugated goat anti-rabbit IgG polyclonal antibody (HA1123), and FITC-conjugated goat anti-mouse IgG polyclonal antibody (HA1128) were bought from HUABIO (Hangzhou, China). d-Luciferin potassium was bought from Dalian Meilun Biotechnology Co. Ltd. (Dalian, China). Purified anti-mouse CD16/32 antibody was bought from Elabscience Biotechnology Co. Ltd. (Wuhan, China). FITC anti-mouse CD3 antibody, phycoerythrin (PE) anti-mouse CD4 antibody, Allophycocyanin (APC) anti-mouse CD8 antibody, PE anti-mouse CD11b antibody, FITC anti-mouse F4/80 antibody, PC5.5 anti-mouse CD86 antibody, APC anti-mouse CD206 antibody, PE anti-mouse CD19 antibody, FITC anti-mouse CD27 antibody, PE-Cy7 anti-mouse IgD antibody, APC anti-mouse IgG antibody, APC anti-mouse CD47 antibody, APC anti-human CD19 antibody, PE anti-human CD10 antibody, FITC anti-mouse B220 antibody, and PE anti-mouse IgM antibody were purchased from BioLegend (California, USA). Mouse total IgG ELISA kit, mouse anti-dsDNA antibody ELISA kit, and mouse ANA ELISA kit were obtained from Ruixin Biological Technology Co. Ltd. (Quanzhou, China). Bovine CII, complete Freund’s adjuvant (CFA), and incomplete Freund’s adjuvant (IFA) were obtained from Chondrex (Washington, USA). OVA (albumin from chicken egg white) was purchased from Sigma-Aldrich (MO, USA). Horseradish peroxidase–conjugated anti-mouse IgG, IgG1, or IgG2a was purchased from Abcam (Cambridge, UK). RPMI 1640 (L210KJ) was purchased from BasalMedia Co. Ltd. (Shanghai, Chinia). Fetal bovine serum (CE000-N031) was purchased from ExCell Biotechnology Co. Ltd. (Suzhou, China). All reagents were of analytical grade.

### Cell culture

Raji cells (catalog no. CTCC-001-0026) and Raji-GFP cells (catalog no. CTCC-DZX-0032) were purchased from Meisen Cell Technology Co. Ltd. (Zhejiang, China). Raji-Luc (catalog no. FH0142) were purchased from Fuheng Cell Center (Shanghai, China). Each cell was cultured in RPMI 1640 culture, with 10% fetal bovine serum and 1% penicillin/streptomycin. The cell culture incubator maintained a temperature of 37°C under a 5% CO_2_ atmosphere.

### Animals and ethics statement

CB-17 SCID (CB17/Icr-*Prkdc*^scid^/IcrlcoCrl) mice were purchased from Beijing Vital River Laboratory Animal Co. Ltd. BALB/c, C57BL/6J, and MRL-*lpr* (MRL/MpJ-Fas*^lpr^*/J) mice were purchased from Beijing SPF Biotechnology Co. Ltd. DBA/1JGpt mice (catalog no. N000219) were purchased from Nanjing GemPharmatech. All mice were housed under specific pathogen–free conditions at 22° ± 1°C and 60 ± 5% relative humidity with a 12-hour light/dark cycle. All animal experiments were approved by the Institutional Animal Care and Ethics Committee of Sichuan University and conducted in the Animal Laboratory of West China School of Pharmacy in Sichuan University [SYXK (Chuan) 2024-0185].

### Synthesis and characterization of anti-CD20 mAb-N_3_

To conjugate azide groups to anti-human CD20 mAb RTX, RTX with the concentration of 5 mg/ml was reduced by 10 equivalents of TCEP in phosphate-buffered saline (PBS; pH 7.4) containing 1 mM EDTA-2Na for 40 min at 37°C to selectively expose the thiol group in the hinge region. After removing excess of TCEP by ultrafiltration [30,000-Da Molecular weight cut off (MWCO); Millipore] with PBS three times, Ellman’s assay was used to quantify the amount of exposed thiol groups on antibodies. The thiol-exposed antibody (RTX-Thiolated rituximab; 5 mg/ml) was then reacted with a 12-fold molar excess of N_3_–PEG-1000–maleimide at room temperature overnight. The fabricated RTX-N_3_ (anti-CD20 mAb-N_3_) was further purified by ultrafiltration with PBS (pH 7.4) washing three times. Conjugation efficiency was determined through dual analytical approaches. Ellman’s assay measured residual thiol groups postconjugation, with the difference between initial exposed and residual thiol counts equating to the number of conjugated N_3_–PEG-1000–maleimide molecules per antibody. In parallel, anti-CD20 mAb-N_3_ was incubated with a 12-fold molar excess of DBCO-Cy5 at 37°C for 1 hour. Following removal of unreacted DBCO-Cy5 by ultrafiltration, the conjugation stoichiometry was calculated on the basis of Cy5 absorbance measurements. Both methods yielded concordant values. Next, the coupling position of N_3_–PEG-1000–maleimide was examined using reducing SDS-PAGE, and the structural integrity of anti-CD20 mAb-N_3_ was examined using nonreducing SDS-PAGE. To synthesize Cy3-labeled and Cy5-labeled anti-CD20 mAb-N_3_, RTX with the concentration of 5 mg/ml was reacted with a twofold molar excess of sulfo-Cy3 NHS ester or sulfo-Cy5 NHS ester in PBS (pH 7.4) for 3 hours at room temperature. After ultrafiltration removal of unconjugated dyes, the obtained Cy3-labeled and Cy5-labeled anti-CD20 mAb-N_3_ was reduced by TCEP to expose hinge-region thiol groups and conjugated with N_3_–PEG-1000–maleimide as described above. For the anti-mouse CD20 mAb MB20-11, synthesis and characterization were performed according to the aforementioned method.

### Preparation and characterization of Erythrocyte-DBCO

For in vitro proof-of-concept demonstration, erythrocyte source of Erythrocyte-DBCO was from C57BL/6 mice. For in vivo validation of NHL, SLE, and RA therapy, Erythrocyte-DBCO was derived from host-matched erythrocytes from CB-17 SCID, MRL-*lpr*, and DBA/1J mouse models, respectively. Whole blood was collected from C57BL/6 mice via the retro-orbital venous plexus into EDTA-2K anticoagulant tubes. After centrifugation at 3000 rpm for 10 min to remove the plasma, the isolated erythrocytes were washed three times with cold PBS (pH 7.4) and stored in Alsever’s solution at 4°C within a week. To verify the feasibility of lipid insertion into red blood cell membranes, isolated erythrocytes (1 × 10^8^ cells) were firstly centrifuged at 3000 rpm for 3 min to remove Alsever’s solution and washed three times with PBS. Erythrocytes were then resuspended in 1 ml of FITC–PEG-2000–DSPE (80 μg/ml in PBS) and incubated at 37°C for 15 min. After centrifugation at 3000 rpm for 3 min, the isolated erythrocytes were washed three times with PBS before confocal visualization. Parallel samples were analyzed for FITC fluorescence by flow cytometry (Beckman Coulter, USA). For the preparation of Erythrocyte-DBCO, isolated erythrocytes (1 × 10^8^ cells) were firstly centrifuged at 2000 rpm for 3 min to remove Alsever’s solution and washed three times with PBS. Erythrocytes were then resuspended in 1 ml of DBCO–PEG-2000–DSPE (80 μg/ml in PBS) and incubated at 37°C for 15 min. After centrifugation at 2000 rpm for 3 min, the isolated Erythrocyte-DBCO were washed three times with PBS.

### Clickable reactivity on anti-CD20 mAb-N_3_–pretargeted B cells and Erythrocyte-DBCO

To investigate surface exposure of clickable azide groups by bifunctional adaptors, Raji cells (2 × 10^5^) were first incubated with Cy3-labeled anti-CD20 mAb-N_3_ (1 μM) for 1 hour at 37°C. Following three washes with cold PBS (pH 7.4) to remove unbound Cy3-labeled anti-CD20 mAb-N_3_, cells were incubated with DBCO-Cy5 (5 μM) for 1 hour at 37°C. After PBS washes, cell nuclei were stained with Hoechst 33342 (5 μg/ml) for 5 min. At the end, cells were washed three times with cold PBS and plated onto sterile 35-mm glass bottom culture dishes before CLSM (Zeiss LSM 800, Germany) visualization. Parallel samples were analyzed for Cy3 and Cy5 fluorescence by flow cytometry. For surface exposure of clickable DBCO groups, Erythrocyte-DBCO (1 × 10^7^ cells) were firstly incubated with N_3_-Cy5 (5 μg/ml in 1 ml of PBS) for 1 hour at 37°C. Following three washes with cold PBS to remove unbound N_3_-Cy5, cells were observed by CLSM and further analyzed through flow cytometry.

### Morphology and biology changes in EryPatch

To evaluate membrane tension, Erythrocyte-DBCO (1 × 10^7^ cells) were resuspend in 1 ml of PBS (pH 7.4) and incubated at 37°C for 0, 1, 3, 6, 12, and 24 hours, respectively. Cells were labeled with the membrane tension probe Flipper-TR according to the manufacturer’s protocol. FLIM (Leica STELLARIS 8 DIVE) was performed using a 488-nm pulsed laser for excitation, and emitted photons were collected through a 600/50-nm bandpass filter. An increase in fluorescence lifetime was interpreted as indicative of elevated membrane tension.

For membrane fluidity, Erythrocyte-DBCO were labeled with DiD fluorescent dye according to fluorescence recovery after photobleaching’s method (*42*). Fluorescence recovery (%) data were collected through CLSM. The higher fluorescence recovery (%) corresponded to greater membrane fluidity.

For the investigation of mechanical fragility, Erythrocyte-DBCO were resuspended in isotonic saline containing 2.5-mm glass shaking beads, followed by orbital shaking at 60 rpm and 37°C for 3 hours. The extent of hemoglobin release was quantified by measuring the absorbance of the supernatant at 545 nm. For osmotic fragility, Erythrocyte-DBCO cells were resuspended in 0.45% saline and incubated at 37°C under shaking for 1 hour. Hemoglobin release in the supernatant was similarly quantified by absorbance measurement at 545 nm.

In addition, Erythrocyte-DBCO were resuspended in 1 ml of PBS and incubated at 37°C for 0 and 24 hours. Cell samples were submitted for label-free proteomics analysis (Shanghai Omics-space Biotech Co. Ltd., Shanghai, China). The analysis included protein extraction, protein digestion, liquid chromatography–tandem mass spectrometry detection, protein quantitation and identification (MaxQuant 1.5.5.1), and bioinformatics analysis.

To evaluate sialic acid levels, Erythrocyte-DBCO were resuspended in 10 mM tris-HCl buffer (pH 7.4) and incubated at 4°C for 1 hour. After centrifugation (9000 rpm at 4°C for 15 min), the mixture was washed three times with PBS to obtain a white membrane suspension. This suspension was then hydrolyzed with 0.1 M H_2_SO_4_ in an 80°C water bath for 1 hour. After cooling to room temperature, the mixture was centrifuged (9000 rpm at 4°C for 15 min), and the supernatant was collected as the test sample. The sialic acid content was quantified by measuring absorbance using an assay kit.

For CD47 expression analysis, Erythrocyte-DBCO were resuspended with APC anti-mouse CD47 antibody (1 μg/ml) at 4°C for 1 hour and washed with PBS. CD47 expression was subsequently analyzed by flow cytometry.

To evaluate ATP levels, Erythrocyte-DBCO were lysed at designated time points. After centrifuging (12000 rpm) for 10 min, supernatant was transferred to a 96-well plate and mixed with ATP detection solution. Luminescence was measured using multimode microplate reader.

### Attachment of B cell and CD20 EryPatch

For the investigation of CD20 EryPatch attachment on Raji cells, Erythrocyte-DBCO were prelabeled with CellTrace Far Red. Raji-GFP cells (2 × 10^5^) were then incubated with anti-CD20 mAb-N_3_ (1 μM) for 1 hour at 37°C. Following three washes with cold PBS to remove unbound anti-CD20 mAb-N_3_, Raji-GFP were incubated with Erythrocyte-DBCO (6 × 10^5^) for 0, 1, 2, and 3 hours at 37°C. Following incubation, cell populations were analyzed by flow cytometry to determine the percentage of double-positive cell clusters.

### Discocyte-to-echinocyte transition of EryPatch on B cells

Raji cells (2 × 10^5^) were treated with anti-CD20 mAb-N_3_ (1 μM) for 1 hour at 37°C, followed by incubation with Erythrocyte-DBCO (6 × 10^5^ cells) for 1, 3, 6, 12, and 24 hours. After the treatments, cells were washed three times with cold PBS, fixed with 3% glutaraldehyde, and observed using CLSM. Parallel samples were captured by SEM (JSM-IT700HR, JEOL) supported by Chengdu Lilai Biotechnology Co. Ltd. (Chengdu, China).

### Preparation and characterization of NP-DBCO

The NP-DBCO was prepared through a nanoprecipitation method (*43*). In detail, 1.0 mg of PLGA, 0.4 mg of DSPE–PEG-2000–DBCO, and 0.2 mg of soybean phospholipid were dissolved in 100 μl of dimethyl sulfoxide. This organic solution was then added dropwise into 2.0 ml of deionized water under continuous stirring for 10 min. The nanoparticle suspension was concentrated with an ultrafiltration tube (30,000 Da) and washed with deionized water to remove organic solvents. Particle size distribution and zeta potential were measured by Zetasizer Nano ZS90 (Malvern, UK). The morphology of NP-DBCO was examined using transmission electron microscopy (Hitachi Model H600, Japan).

### Spatial distribution and cross-linking of CD20 receptors

For spatial distribution of CD20 receptors on Raji cell surface, Raji cells (2 × 10^5^) were treated with (i) CD20 mAb [Cy3-labeled anti-CD20 mAb-N_3_ (1 μM, 1 hour) → fresh RPMI 1640 (24 hours)], (ii) CD20 NP [Cy3-labeled anti-CD20 mAb-N_3_ (1 μM, 1 hour) → FITC-labeled NP-DBCO (PLGA: 0.5 mg, 24 hours)], (iii) fixed CD20 EryPatch [Cy3-labeled anti-CD20 mAb-N_3_ (1 μM, 1 hour) → fixed FITC-labeled Erythrocyte-DBCO (6 × 10^5^ cells, 24 hours)], or (iv) CD20 EryPatch [Cy3-labeled anti-CD20 mAb-N_3_ (1 μM, 1 hour) → FITC-labeled Erythrocyte-DBCO (6 × 10^5^ cells, 24 hours)] at 37°C. Following the treatments, cells were washed three times with cold PBS, stained with 4′,6-diamidino-2-phenylindole, and observed using CLSM.

For the CD20 EryPatch–induced clustering FRET experiments, Cy3- and Cy5-labeled anti-CD20 mAb-N_3_ were synthesized as described above. Raji cells (2 × 10^5^) were treated with (i) CD20 mAb [equimolar Cy3- and Cy5-labeled anti-CD20 mAb-N_3_ (0.5 μM each, 1 hour) → fresh RPMI 1640 (24 hours)], (ii) CD20 NP [equimolar Cy3- and Cy5-labeled anti-CD20 mAb-N_3_ (0.5 μM each, 1 hour) → NP-DBCO (PLGA: 0.5 mg, 24 hours)], (iii) fixed CD20 EryPatch [equimolar Cy3- and Cy5-labeled anti-CD20 mAb-N_3_ (0.5 μM each, 1 hour) → fixed Erythrocyte-DBCO (6 × 10^5^ cells, 24 hours)], or (iv) CD20 EryPatch [equimolar Cy3- and Cy5-labeled anti-CD20 mAb-N_3_ (0.5 μM each, 1 hour) → Erythrocyte-DBCO (6 × 10^5^ cells, 24 hours)] at 37°C. After incubation, cells were analyzed using FLIM program by Leica STELLARIS 8 DIVE for FRET emission. Fluorescence lifetime was performed using a 488-nm pulsed laser for excitation, and emitted photons were collected through a 530/30-nm bandpass filter. The FRET efficiency (E) is calculated as the following ratio: $E=1-\frac{\tau_{\mathrm{DA}}}{\tau_{D}}$, where $\tau_{D}$ is the unquenched donor lifetime and $\tau_{\mathrm{DA}}$ is the quenched donor lifetime in each defined region of interest. Elevated FRET efficiency corresponded to stronger CD20 receptor cross-linking.

Membrane tension in Raji cells was measured using the Flipper-TR probe as described previously. For the evaluation of calcium influx, Raji cells (2 × 10^5^) were firstly loaded with intracellular calcium indicator Fluo-4 AM (5 μM) for 30 min at 37°C and resuspended in RPMI 1640 containing 2.5 mM Ca2^+^. Following indicated treatments, samples were analyzed by flow cytometry to quantify Fluo-4 AM fluorescence intensity as a measure of calcium influx.

### Downstream apoptotic signaling evaluation

In vitro apoptosis analysis of CD20 EryPatch was quantified using Annexin V–FITC/PI Apoptosis Detection Kit. Raji cells (2 × 10^5^) were treated with (i) CD20 mAb [anti-CD20 mAb-N_3_ (1 μM, 1 hour) → fresh RPMI 1640 (24 hours)], (ii) CD20 NP [anti-CD20 mAb-N_3_ (1 μM, 1 hour) → NP-DBCO (PLGA: 0.5 mg, 24 hours)], (iii) fixed CD20 EryPatch [anti-CD20 mAb-N_3_ (1 μM, 1 hour) → fixed Erythrocyte-DBCO (6 × 10^5^ cells, 24 hours)], or (iv) CD20 EryPatch [anti-CD20 mAb-N_3_ (1 μM, 1 hour) → Erythrocyte-DBCO (6 × 10^5^ cells, 24 hours)] at 37°C. After incubation, cells were washed with PBS and stained with Annexin V–FITC and PI in dark for 15 min according to the manufacturer’s protocol. Apoptosis levels were then quantified by flow cytometry.

To detect Bcl-2 and Bax level, cells received indicated treatments were fixed with 4% paraformaldehyde for 15 min at room temperature and permeabilized with 90% methanol for 30 min on ice. Subsequently, cells were incubated with Bcl-2 mAb (1 μg/ml) or Bax mAb (1 μg/ml) in 3% bovine serum albumin (BSA) buffer for 1 hour at 4°C. After primary antibody staining, samples treated with Bcl-2 mAb were incubated with iFluor 647–conjugated goat anti-rabbit IgG polyclonal antibody (1:1000), and samples treated with Bax mAb were incubated with FITC-conjugated goat anti-mouse IgG polyclonal antibody (1:1000). Following three washes with PBS, fluorescence intensity was analyzed by flow cytometry.

JC-1 mitochondrial membrane potential probe was used to evaluate the extent of mitochondrial depolarization. Following the indicated treatments, cells were washed with PBS and stained with JC-1 (500 μl of PBS; 1 μM) for 30 min at 37°C. The mixture was washed twice with PBS and resuspended in 400 μl of PBS and analyzed by flow cytometry.

For cytochrome c release detection, samples received indicated treatments were fixed with 4% paraformaldehyde for 15 min at room temperature, permeabilized with 90% methanol for 30 min on ice, and incubated with anti–cytochrome c mAb (0.5 μg/ml) in PBS (3% BSA) for 1 hour at 4°C. After washing, cells were stained with FITC-conjugated goat anti-mouse IgG antibody (1:1000) for 1 hour at 4°C. Fluorescence was quantified by flow cytometry.

Caspase-3 activity was evaluated using caspase-3 activity and mitochondrial membrane potential detection kit for live cell. After indicated treatments, cells were washed with PBS and incubated with 200 μl of caspase-3 working solution at room temperature for 30 min. Cells were then washed with PBS, resuspended in 400 μl of PBS, and analyzed by flow cytometry.

### Erythrophagocytosis of B cells

BMDMs were isolated and cultured as previously described (*44*). BMDMs were labeled with DiD fluorescent dye (2 μg/ml) for 30 min at 37°C, washed three times with PBS, and designated as BMDM-DiD. Raji cells (1 × 10^5^) were incubated with anti-CD20 mAb-N_3_ (1 μM) for 1 hour at 37°C, followed by incubation with (i) erythrocytes (6 × 10^5^ cells) and (ii) Erythrocyte-DBCO (6 × 10^5^ cells) for 24 hours. BMDM-DiD (3 × 10^5^ cells) were then added to the coculture system and further incubated for 4 hours at 37°C. Phagocytosis by BMDM was subsequently quantified by flow cytometry.

### Pharmacokinetics of CD20 EryPatch

Eight-week-old male CB-17 SCID mice (*n* = 5) were intravenously injected with Cy5-labeled anti-CD20 mAb-N_3_ (1 nmol). Five hours later, Erythrocyte-DBCO (1 × 10^7^ cells) were administered intravenously. At predetermined time intervals, 10 μl of blood samples was collected from tail vein. The fluorescence intensity of Cy5 in each sample was measured using a multimode microplate reader at excitation wavelength of 650 nm and emission wavelength of 670 nm. The blood concentration of anti-CD20 mAb-N_3_ was determined on the basis of a standard curve prepared with known concentrations of Cy5.

For pharmacokinetic analysis of Erythrocyte-DBCO, 8-week-old male CB-17 SCID mice (*n* = 5) received anti-CD20 mAb-N_3_ (1 nmol) intravenously, followed 5 hours later by biotin-labeled Erythrocyte-DBCO (1 × 10^7^ cells). Blood was collected and lysed at designated time points. After centrifuging (12,000 rpm) for 10 min, supernatant was analyzed by Biotin Quantitative Assay Kit to determine biotin concentration. For biodistribution assessment, major organs (heart, liver, spleen, lung, and kidney) were harvested 24 hours postinjection, homogenized, and analyzed for biotin content using the same kit.

To verify the bioorthogonal conjugation in vivo, disseminated NHL models were established in 8-week-old male CB-17 SCID mice by intravenous injection of 4 × 10^6^ Raji-Luc cells. One week later, mice received Cy5-labeled anti-CD20 mAb-N_3_ (1 nmol) intravenously, followed 5 hours later by an injection of either Cy5.5-labeled Erythrocyte-DBCO or Cy5.5-labeled native erythrocytes (1 × 10^7^ cells). Blood samples collected 3 hours after the second injection were analyzed by IVIS spectrum imaging [Cy5: Excitation wavelength (Ex)/ Emission wavelength (Em) of 620/660 nm; Cy5.5: Ex/Em of 660/710 nm; FRET: Ex/Em of 620/710 nm].

To quantify binding efficiency under physiological flow, a circulating system mimicking blood flow was established using a peristaltic pump (flow velocity: 20 ml/min; circuit volume: 2 ml; 37°C). Raji-GFP cells, pretargeted with anti-CD20 mAb-N_3_ (1 nmol), were circulated with CellTrace Far Red–labeled Erythrocyte-DBCO at effector-to-target ratios of 1:1, 3:1, and 5:1. Samples were collected at 0, 1, 2, and 3 hours for flow cytometric analysis. For morphological assessment, cells were fixed at 3, 6, 12, and 24 hours and processed for SEM. Parallel samples were stained with APC–anti-mouse CD47 antibody (1 μg/ml) for flow cytometric analysis of CD47 expression over time.

### In vivo antilymphoma efficacy study

Eight-week-old male CB-17 SCID mice were intravenously injected with 4 × 10^6^ Raji-Luc cells in 200 μl of PBS via the tail vein on day 0. On days 8, 10, and 12, 100 μl of saline, anti-human CD20 mAb RTX (1 nmol), or CD20 EryPatch [RTX-N_3_ (1 nmol) → Erythrocyte-DBCO (1 × 10^7^ cells, with a 5-hour interval)] was given intravenously to the randomly divided mice groups (*n* = 5 or 10). For macrophage ablation studies, mice were injected intraperitoneally with clodronate liposomes in accordance with the manufacturer’s recommended dosage and schedule (200 μl/20 g), with liposomes administered 7 days before CD20 EryPatch treatment to ensure effective macrophage clearance. Tumor progression was monitored using the IVIS spectrum imaging system (PerkinElmer Lumina Series III, USA) at predetermined time points. Body weight and survival were recorded throughout the study, with the experimental endpoint defined as the onset of hindlimb paralysis or over 20% body weight loss. After mice were euthanized, fresh femurs from both hindlimbs were purged with 5 ml of PBS to obtain BM cells. The cell suspension was filtered through a 70-μm nylon strainer and incubated with ACK lysis buffer for 5 min at 4°C to remove red blood cells. After three washes with cold PBS, cells were incubated with anti-CD16/32 antibody to block nonspecific interaction with Fc receptors for 30 min at 4°C. Then, cells were resuspended in 100 μl of PBS and stained with APC anti-human CD19 and PE anti-human CD10 antibodies for 30 min at 4°C in the dark and then washed with PBS before flow cytometry analysis. Raji cells were used as a positive control for gating. Samples were analyzed by flow cytometry to quantify metastatic Raji cells (CD10^+^CD19^+^) in the BM. The hindlimbs were collected at the endpoint and scanned using a micro-CT system (Bruker, SkyScan 1276, Germany). 3D reconstructions were generated to evaluate bone microstructure and quantify histomorphometric parameters, including BV/TV, Tb.Th, and Tb.Sp, for assessment of lymphoma-induced bone damage.

### In vivo therapeutic efficacy in SLE model

Thirteen-week-old female MRL-*lpr* mice were randomly divided into three groups (*n* = 5) and received weekly intravenous administrations of saline, anti-mouse CD20 mAb MB20-11 (2 nmol), or CD20 EryPatch [MB20-11-N_3_ (2 nmol) → Erythrocyte-DBCO (1 × 10^7^ erythrocytes, with a 5-hour interval)] for four consecutive weeks, and age-matched C57BL/6J mice without recessive *lpr* gene mutation served as healthy control. Urine protein levels and body weight were monitored throughout the study. At 17 weeks of age, spleen, BM, blood, and lymph nodes were collected for B cell depletion analysis. Single-cell suspensions were prepared and stained with FITC anti-mouse B220 and PE anti-mouse IgM antibodies for 30 min at 4°C in the dark and then washed with PBS before flow cytometry analysis. Samples were analyzed by flow cytometry to quantify mature B cells (IgM^+^B220^high^). Lymph nodes and spleens were dissected and weighed to assess organ index. Serum was isolated for measurement of total IgG, anti-dsDNA antibodies, and ANA using ELISA kits. Kidney tissues were harvested and subjected to H&E staining for histopathological examination, while frozen sections were incubated with anti-mouse IgG and anti-C3 antibodies for immunofluorescence evaluation of glomerular deposition (Hubei BIOSSCI Biotech Co. Ltd).

### In vivo therapeutic efficacy in CIA model

The autoimmune arthritis model was established by immunization with an emulsion of Freund’s adjuvant and CII (*45*). Specifically, 8-week-old male DBA/1J mice were injected with bovine CII emulsified in CFA, followed by boosting 21 days later with bovine CII emulsified in IFA. On days 20, 22, and 24, 100 μl of saline, anti-mouse CD20 mAb MB20-11 (1 nmol), or CD20 EryPatch [MB20-11-N_3_ (1 nmol) → Erythrocyte-DBCO (1 × 10^7^ erythrocytes, with a 5-hour interval)] was given intravenously to the randomly divided mice groups. For macrophage ablation studies, clodronate liposome was injected intraperitoneally 7 days before CD20 EryPatch treatment with recommended dosage (200 μl/20 g). Paw thickness and clinical arthritis scores were evaluated throughout the treatment period to record the development of arthritis. Arthritis severity was graded on a 0-to-4 scale for each paw by the following scale: absence of inflammation = 0, paw with detectable swelling in a single digit = 1, paw with swelling in more than one digit = 2, paw with swelling of all digits and instep = 3, and severe swelling of the paw and ankle = 4.

On days 0, 18, 24, and 40, spleen, BM, blood, and lymph nodes were collected for B cell depletion analysis. On day 40, ankle joint tissues were harvested and scanned using micro-CT to reconstruct the 3D structure of hind ankle joints and evaluate the histomorphometric characteristics. The knee and ankle joints were decalcified after fixation. The samples were cross sectioned into 5-μm-thick slices. Tissue slices were stained with H&E, Safranin O–Fast Green, and Masson’s trichrome for histological evaluation. Knee frozen sections were incubated with anti-mouse F4/80, CD86, and CD206 antibodies for immunofluorescence evaluation of macrophage polarization (Hubei BIOSSCI Biotech Co. Ltd).

To explore the mechanism behind the therapeutic efficacy of CD20 EryPatch, synovial tissues from healthy mice, RA mice, and RA mice treated with MB20-11 or CD20 EryPatch were analyzed by scRNA-seq on the BGI DNBSEQ-T7 PE100. The sequencing and bioinformatics analysis were provided by OE Biotech Co. Ltd. (Shanghai, China).

### Biosafety of CD20 EryPatch

Eight-week-old male DBA1/J mice (*n* = 5) received three administrations of 100 μl of PBS or MB20-11-N_3_ (1 nmol) and Erythrocyte-DBCO (1 × 10^7^ cells) 5 hours later on days 0, 2, and 4. On day 10, mice were euthanized, and whole blood and serum samples were collected for complete blood count and blood biochemistry analysis. Spleen weights were recorded, and major organs were harvested for H&E staining.

For T cell activation analysis, single-cell suspensions from the spleen and lymph nodes were incubated with anti-CD16/32 antibody to block nonspecific interaction with Fc receptors for 30 min at 4°C. Then, cells were resuspended in 100 μl of PBS and stained with FITC anti-mouse CD3, PE anti-mouse CD4, and APC anti-mouse CD8 antibodies for 30 min at 4°C in the dark. After washing, samples were analyzed by flow cytometry to quantify proportions of CD3^+^CD8^+^ and CD3^+^CD4^+^ T cells.

For macrophage polarization analysis, cells were resuspended in 100 μl of PBS and stained with PE anti-mouse CD11b, FITC anti-mouse F4/80, PC5.5 anti-mouse CD86, and APC anti-mouse CD206 antibodies for 30 min at 4°C in the dark. After washing, samples were analyzed by flow cytometry to quantify mean fluorescence intensity of CD86 and CD206 in CD11b^+^F4/80^+^ cells.

### Assessment of B cell reconstitution and immune competence

Eight-week-old male DBA1/J mice (*n* = 5) received three administrations of MB20-11-N_3_ (1 nmol) and Erythrocyte-DBCO (1 × 10^7^ cells) 5 hours later on days −20, −18, and −16. Upon confirmation of B cell recovery (day 0), mice were immunized subcutaneously with 10 μg of OVA emulsified in 0.2 mg of alum on day 1. A booster immunization was administered on day 15. Blood samples were collected on days 8 and 15 for serological analysis. Serum levels of OVA-specific IgG, IgG1, and IgG2a were measured by ELISA according to an established protocol (*46*). Titers were defined as the reciprocal of the highest serum dilution yielding an optical density at 450 nm value 2.5-fold above the mean background. On days 0 and 15, mice were euthanized and spleen, lymph nodes, BM, and blood were harvested for B cell phenotyping analysis. Briefly, Fc-blocked single-cell suspensions were resuspended in 100 μl PBS and stained with PE anti-mouse CD19, FITC anti-mouse CD27, PE-Cy7 anti-mouse IgD, and APC anti-mouse IgG antibodies for 30 min at 4°C in the dark. After washing, samples were analyzed by flow cytometry to quantify proportions of B cell subsets.

### Statistical analysis

Statistical data were analyzed using the Graphpad Prism 10 software and presented as mean ± SD. Statistical significance was calculated via an unpaired Student’s *t* test for two-group comparisons. For multiple comparisons, statistical differences were determined using one-way analysis of variance (ANOVA), followed by Tukey’s multiple comparisons. A *P* value of less than 0.05 was considered statistically significant.

## References

[1] M. F. Sánchez, R. Tampé, Ligand-independent receptor clustering modulates transmembrane signaling: A new paradigm. Trends Biochem. Sci. 48, 156–171 (2023). 36115755 10.1016/j.tibs.2022.08.002

[2] K. Zhang, H. Gao, R. Deng, J. Li, Emerging applications of nanotechnology for controlling cell-surface receptor clustering. Angew. Chem. Int. Ed. Engl. 58, 4790–4799 (2019). 30328227 10.1002/anie.201809006

[3] B. Belardi, S. M. Son, J. H. Felce, M. L. Dustin, D. A. Fletcher, Cell–cell interfaces as specialized compartments directing cell function. Nat. Rev. Mol. Cell Biol. 21, 750–764 (2020). 33093672 10.1038/s41580-020-00298-7

[4] L. L. Kiessling, J. E. Gestwicki, L. E. Strong, Synthetic multivalent ligands as probes of signal transduction. Angew. Chem. Int. Ed. Engl. 45, 2348–2368 (2006). 16557636 10.1002/anie.200502794 PMC2842921

[5] L. Li, J. Yang, J. Wang, J. Kopeček, Amplification of CD20 cross-linking in rituximab-resistant B-lymphoma cells enhances apoptosis induction by drug-free macromolecular therapeutics. ACS Nano 12, 3658–3670 (2018). 29595951 10.1021/acsnano.8b00797 PMC5916500

[6] T. Chu, J. Yang, R. Zhang, M. Sima, J. Kopeček, Cell surface self-assembly of hybrid nanoconjugates via oligonucleotide hybridization induces apoptosis. ACS Nano 8, 719–730 (2014). 24308267 10.1021/nn4053827 PMC3908873

[7] L. Li, J. Wang, Y. Li, D. C. Radford, J. Yang, J. Kopeček, Broadening and enhancing functions of antibodies by self-assembling multimerization at cell surface. ACS Nano 13, 11422–11432 (2019). 31553883 10.1021/acsnano.9b04868 PMC6812323

[8] L. Li, J. Yang, S. Soodvilai, J. Wang, P. Opanasopit, J. Kopeček, Drug-free albumin-triggered sensitization of cancer cells to anticancer drugs. J. Control. Release 293, 84–93 (2019). 30465822 10.1016/j.jconrel.2018.11.015 PMC6317733

[9] S. Bi, W. Chen, Y. Fang, Y. Wang, Q. Zhang, H. Guo, H. Ju, Y. Liu, Cancer cell-selective membrane receptor clustering driven by VEGF secretion for in vivo therapy. J. Am. Chem. Soc. 145, 5041–5052 (2023). 36815672 10.1021/jacs.2c10428

[10] Y. Wang, I. Baars, F. Fördös, B. Högberg, Clustering of death receptor for apoptosis using nanoscale patterns of peptides. ACS Nano 15, 9614–9626 (2021). 34019379 10.1021/acsnano.0c10104 PMC8223489

[11] L. Zhang, Y. Fang, J. Yang, J. Kopeček, Drug-free macromolecular therapeutics: Impact of structure on induction of apoptosis in Raji B cells. J. Control. Release 263, 139–150 (2017). 28024916 10.1016/j.jconrel.2016.12.025 PMC5538940

[12] J. Yang, L. Li, J. Kopeček, Biorecognition: A key to drug-free macromolecular therapeutics. Biomaterials 190-191, 11–23 (2019). 30391799 10.1016/j.biomaterials.2018.10.007

[13] M. Zhou, C. Liu, B. Li, J. Li, P. Zhang, Y. Huang, L. Li, Cell surface patching via CXCR4-targeted nanothreads for cancer metastasis inhibition. Nat. Commun. 15, 2763 (2024). 38553476 10.1038/s41467-024-47111-z PMC10980815

[14] J. Shi, C. Liu, J. Liu, Y. Yan, F. Wang, S. Yan, Y. Xiang, M. Zhou, Y. Xu, L. Li, CXCR4 clustering induced by polymeric nanothreads impedes cancer cell metastasis via PIEZO1-mediated mechanotransduction. Adv. Healthc. Mater. 14, e2501072 (2025). 40567012 10.1002/adhm.202501072

[15] L. Pan, T. Fu, W. Zhao, L. Zhao, W. Chen, C. Qiu, W. Liu, Z. Liu, A. Piai, Q. Fu, S. Chen, H. Wu, J. J. Chou, Higher-order clustering of the transmembrane anchor of DR5 drives signaling. Cell 176, 1477–1489.e14 (2019). 30827683 10.1016/j.cell.2019.02.001 PMC6529188

[16] M. Stockfelt, Y. K. O. Teng, E. M. Vital, Opportunities and limitations of B cell depletion approaches in SLE. Nat. Rev. Rheumatol. 21, 111–126 (2025). 39815102 10.1038/s41584-024-01210-9

[17] D. S. W. Lee, O. L. Rojas, J. L. Gommerman, B cell depletion therapies in autoimmune disease: Advances and mechanistic insights. Nat. Rev. Drug Discov. 20, 179–199 (2021). 33324003 10.1038/s41573-020-00092-2 PMC7737718

[18] S. Prakash, N. Kumbhojkar, A. Lu, N. Kapate, V. C. Suja, K. S. Park, L. L. Wang, S. Mitragotri, Polymer micropatches as natural killer cell engagers for tumor therapy. ACS Nano 17, 15918–15930 (2023). 37565806 10.1021/acsnano.3c03980

[19] T. Chu, J. Kopeček, Drug-free macromolecular therapeutics - A new paradigm in polymeric nanomedicines. Biomater. Sci. 3, 908–922 (2015). 26191406 10.1039/C4BM00442F PMC4505834

[20] L. Li, J. Yang, J. Wang, J. Kopeček, Drug-free macromolecular therapeutics induce apoptosis via calcium influx and mitochondrial signaling pathway. Macromol. Biosci. 18, 201700196 (2018). 10.1002/mabi.201700196 PMC5912161 28805013

[21] K. R. Shankland, J. O. Armitage, B. W. Hancock, Non-Hodgkin lymphoma. Lancet 380, 848–857 (2012). 22835603 10.1016/S0140-6736(12)60605-9

[22] W. Alduaij, T. M. Illidge, The future of anti-CD20 monoclonal antibodies: Are we making progress? Blood 117, 2993–3001 (2011). 21209380 10.1182/blood-2010-07-298356

[23] J. Jensen, E. Balish, Enhancement of susceptibility of CB-17 mice to systemic candidiasis by poly(I. C)-induced interferon. Infect. Immun. 61, 3530–3532 (1993). 8335384 10.1128/iai.61.8.3530-3532.1993 PMC281033

[24] T. Chu, R. Zhang, J. Yang, M. P. Chao, P. J. Shami, J. Kopeček, A two-step pretargeted nanotherapy for CD20 cross-linking may achieve superior anti-lymphoma efficacy to rituximab. Theranostics 5, 834–846 (2015). 26000056 10.7150/thno.12040 PMC4440441

[25] Y. He, C. Cheng, Y. Liu, F. Chen, Y. Chen, C. Yang, Z. Zhao, J. Dawulieti, Z. Shen, Y. Zhang, J. Du, S. Guan, D. Shao, Intravenous senescent erythrocyte vaccination modulates adaptive immunity and splenic complement production. ACS Nano 18, 470–482 (2024). 38146673 10.1021/acsnano.3c07943

[26] M. Aringer, K. Costenbader, D. Daikh, R. Brinks, M. Mosca, R. Ramsey-Goldman, J. S. Smolen, D. Wofsy, D. T. Boumpas, D. L. Kamen, D. Jayne, R. Cervera, N. Costedoat-Chalumeau, B. Diamond, D. D. Gladman, B. Hahn, F. Hiepe, S. Jacobsen, D. Khanna, K. Lerstrøm, E. Massarotti, J. McCune, G. Ruiz-Irastorza, J. Sanchez-Guerrero, M. Schneider, M. Urowitz, G. Bertsias, B. F. Hoyer, N. Leuchten, C. Tani, S. K. Tedeschi, Z. Touma, G. Schmajuk, B. Anic, F. Assan, T. M. Chan, A. E. Clarke, M. K. Crow, L. Czirják, A. Doria, W. Graninger, B. Halda-Kiss, S. Hasni, P. M. Izmirly, M. Jung, G. Kumánovics, X. Mariette, I. Padjen, J. M. Pego-Reigosa, J. Romero-Diaz, Í. R. F. Fernández, R. Seror, G. H. Stummvoll, Y. Tanaka, M. G. Tektonidou, C. Vasconcelos, E. M. Vital, D. J. Wallace, S. Yavuz, P. L. Meroni, M. J. Fritzler, R. Naden, T. Dörner, S. R. Johnson, 2019 European League Against Rheumatism/American College of Rheumatology classification criteria for systemic lupus erythematosus. Arthritis Rheumatol. 71, 1400–1412 (2019). 31385462 10.1002/art.40930 PMC6827566

[27] A. N. Theofilopoulos, F. J. Dixon, Murine models of systemic lupus erythematosus. Adv. Immunol. 37, 269–390 (1985). 3890479 10.1016/s0065-2776(08)60342-9

[28] J. Wang, Y. Li, L. Li, J. Yang, J. Kopeček, Exploration and evaluation of therapeutic efficacy of drug-free macromolecular therapeutics in collagen-induced rheumatoid arthritis mouse model. Macromol. Biosci. 20, e1900445 (2020). 32196951 10.1002/mabi.201900445 PMC7549750

[29] J. Lichtnekert, H. J. Anders, Lupus nephritis-related chronic kidney disease. Nat. Rev. Rheumatol. 20, 699–711 (2024). 39317803 10.1038/s41584-024-01158-w

[30] J. C. W. Edwards, G. Cambridge, B-cell targeting in rheumatoid arthritis and other autoimmune diseases. Nat. Rev. Immunol. 6, 394–403 (2006). 16622478 10.1038/nri1838

[31] S. Wang, Y. Zhang, Y. Wang, Y. Yang, S. Zhao, T. Sheng, Y. Zhang, Z. Gu, J. Wang, J. Yu, An in situ dual-anchoring strategy for enhanced immobilization of PD-L1 to treat autoimmune diseases. Nat. Commun. 14, 6953 (2023). 37907476 10.1038/s41467-023-42725-1 PMC10618264

[32] T. L. Hunter, Y. J. Bao, Y. Zhang, D. Matsuda, R. Riener, A. Wang, J. J. Li, F. Soldevila, D. S. H. Chu, D. P. Nguyen, Q. C. Yong, B. Ross, M. Nguyen, J. Vestal, S. Roberts, D. Galvan, J. B. Vega, D. Jhung, M. Butcher, J. Nguyen, S. Zhang, C. Fernandez, J. Chen, C. Herrera, Y. Kuo, E. M. Pica, G. Mondal, A. L. Mammen, J. Scholler, S. P. Tanis, S. A. Sievers, A. M. Frantz, G. B. Adams, L. Shawver, R. Farzaneh-Far, M. Rosenzweig, P. P. Karmali, A. I. Bot, C. H. June, H. Aghajanian, In vivo CAR T cell generation to treat cancer and autoimmune disease. Science 388, 1311–1317 (2025). 40536974 10.1126/science.ads8473

[33] Y. Huang, X. Nie, X. Liu, Y. Liu, H. Yu, X. Gao, Development of a highly-efficient erythrocyte-drug covalent conjugation platform and its use in treating thrombotic disorders. Cell Res. 33, 887–890 (2023). 37666976 10.1038/s41422-023-00868-2 PMC10624666

[34] C. Wang, X. Sun, L. Cheng, S. Yin, G. Yang, Y. Li, Z. Liu, Multifunctional theranostic red blood cells for magnetic-field-enhanced in vivo combination therapy of cancer. Adv. Mater. 26, 4794–4802 (2014). 24838472 10.1002/adma.201400158

[35] D. Liu, P. Guo, C. McCarthy, B. Wang, Y. Tao, D. Auguste, Peptide density targets and impedes triple negative breast cancer metastasis. Nat. Commun. 9, 2612 (2018). 29973594 10.1038/s41467-018-05035-5 PMC6031661

[36] R. S. Riley, E. S. Day, Frizzled7 antibody-functionalized nanoshells enable multivalent binding for Wnt signaling inhibition in triple negative breast cancer cells. Small 13, 201700544 (2017). 10.1002/smll.201700544 PMC5545881 28544579

[37] N. Kumbhojkar, S. Prakash, T. Fukuta, K. Adu-Berchie, N. Kapate, R. An, S. Darko, V. C. Suja, K. S. Park, A. P. Gottlieb, M. G. Bibbey, M. Mukherji, L. L. Wang, D. J. Mooney, S. Mitragotri, Neutrophils bearing adhesive polymer micropatches as a drug-free cancer immunotherapy. Nat. Biomed. Eng. 8, 579–592 (2024). 38424352 10.1038/s41551-024-01180-z

[38] K. Zhang, Y. Ma, D. Wang, J. Liu, J. An, Y. Li, C. Ma, Y. Pei, Z. Zhang, J. Liu, J. Shi, In vivo activation of T-cell proliferation by regulating cell surface receptor clustering using a pH-driven interlocked DNA nano-spring. Nano Lett. 22, 1937–1945 (2022). 35225623 10.1021/acs.nanolett.1c04562

[39] L. Li, J. Yin, W. Ma, L. Tang, J. Zou, L. Yang, T. Du, Y. Zhao, L. Wang, Z. Yang, C. Fan, J. Chao, X. Chen, A DNA origami device spatially controls CD95 signalling to induce immune tolerance in rheumatoid arthritis. Nat. Mater. 23, 993–1001 (2024). 38594486 10.1038/s41563-024-01865-5

[40] Y. Li, F. Raza, Y. Liu, Y. Wei, R. Rong, M. Zheng, W. Yuan, J. Su, M. Qiu, Y. Li, F. Raza, Y. Liu, Y. Wei, R. Rong, M. Zheng, W. Yuan, J. Su, M. Qiu, Clinical progress and advanced research of red blood cells based drug delivery system. Biomaterials 279, 121202 (2021). 34749072 10.1016/j.biomaterials.2021.121202

[41] A. Y. S. Lee, CD20^+^ T cells: An emerging T cell subset in human pathology. Inflamm. Res. 71, 1181–1189 (2022). 35951029 10.1007/s00011-022-01622-x PMC9616751

[42] A. B. Leshem, S. Sloan-Dennison, T. Massarano, S. Ben-David, D. Graham, K. Faulds, H. E. Gottlieb, J. H. Chill, A. Lampel, Biomolecular condensates formed by designer minimalistic peptides. Nat. Commun. 14, 421 (2023). 36702825 10.1038/s41467-023-36060-8 PMC9879991

[43] P. Zhang, B. Li, Z. Wang, J. Li, F. Wang, J. Kong, Z. Zhou, Y. Huang, L. Li, Durable attenuation of tumor pH-platelet linkage reinstates bioorthogonal targeting of residual tumors post-debulking. ACS Nano 18, 4520–4538 (2024). 38270077 10.1021/acsnano.3c11536

[44] M. Li, M. Li, Y. Yang, Y. Liu, H. Xie, Q. Yu, L. Tian, X. Tang, K. Ren, J. Li, Z. Zhang, Q. He, Remodeling tumor immune microenvironment via targeted blockade of PI3K-γ and CSF-1/CSF-1R pathways in tumor associated macrophages for pancreatic cancer therapy. J. Control. Release 321, 23–35 (2020). 32035193 10.1016/j.jconrel.2020.02.011

[45] D. D. Brand, K. A. Latham, E. F. Rosloniec, Collagen-induced arthritis. Nat. Protoc. 2, 1269–1275 (2007). 17546023 10.1038/nprot.2007.173

[46] X. Hong, X. Zhong, G. Du, Y. Hou, Y. Zhang, Z. Zhang, T. Gong, L. Zhang, X. Sun, The pore size of mesoporous silica nanoparticles regulates their antigen delivery efficiency. Sci. Adv. 6, eaaz4462 (2020). 32596445 10.1126/sciadv.aaz4462 PMC7304990

