# Supplementary material for: Erythrocyte patch for enhanced B cell depletion therapy
Source: Sci Adv. 2026 May 20;12(21):eaed3138. doi: 10.1126/sciadv.aed3138 (PMC13189104; doi:10.1126/sciadv.aed3138)
Supplement: Supplementary file 1 — Figs. S1 to S35 [file sciadv.aed3138_sm.pdf]

Science Advances
AAAS

Supplementary Materials for

Erythrocyte patch for enhanced B cell depletion therapy

Jiaqi Liu *et al.*

Corresponding author: Lian Li, liliantripple@163.com

Sci. Adv. **12**, eaed3138 (2026)
DOI: 10.1126/sciadv.aed3138

This PDF file includes:

Figs. S1 to S35

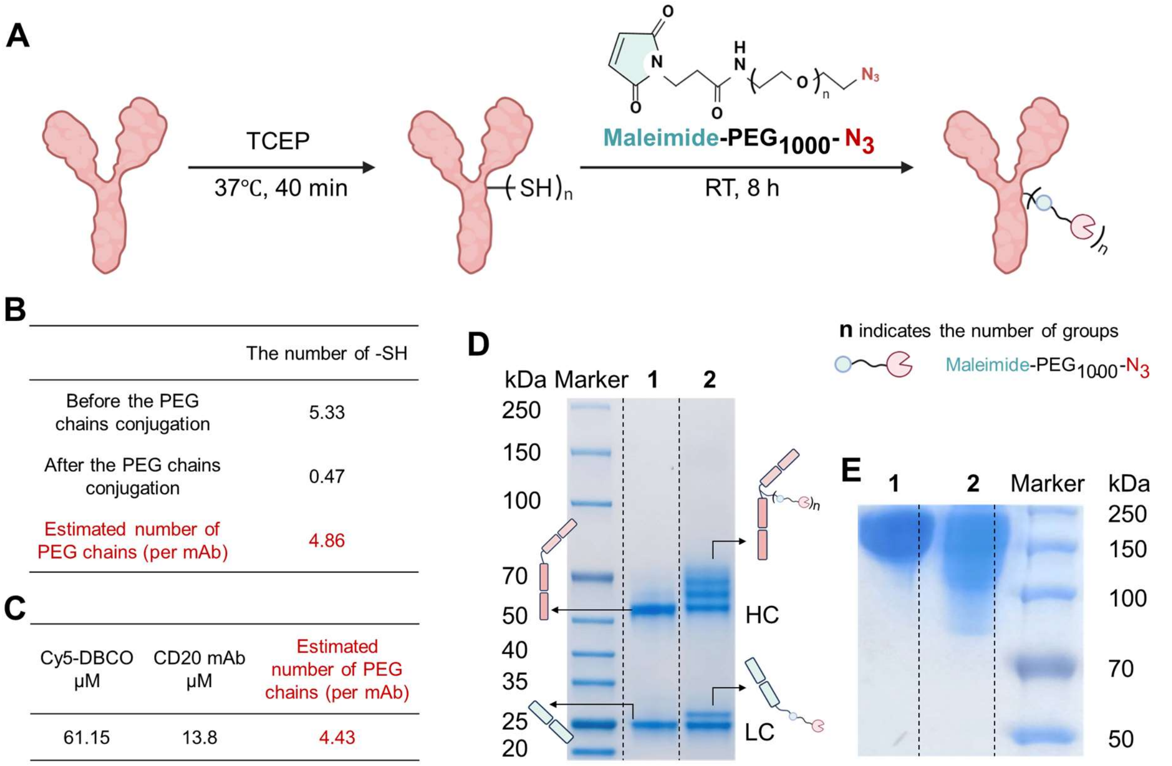

Fig. S1. Synthesis and Characterization of Anti-CD20 mAb-N_3. (**A**) Synthesis route of anti-CD20 mAb-N_3 including two steps: a, TCEP reduction exposes hinge-region thiols (-SH); b, Maleimide-PEG_{1000}-N_3 are conjugated to mAbs via maleimide-thiol chemistry. (**B**) Number of Maleimide-PEG_{1000}-N_3 per mAb estimated from the change in free -SH before and after step b. (**C**) Number of Maleimide-PEG_{1000}-N_3 per mAb estimated via click chemistry binding between N_3 and Cy5-DBCO. (**D**) Reducing SDS-PAGE (Coomassie-stained) showing Maleimide-PEG_{1000}-N_3 conjugation to mAb heavy chains (HC) and a subset of light chains (LC). (**E**) Non-reducing SDS-PAGE (Coomassie-stained) confirming preserved mAb integrity (~150 kDa) post-conjugation. Panel 1, anti-CD20 mAb. Panel 2, anti-CD20 mAb-N_3.

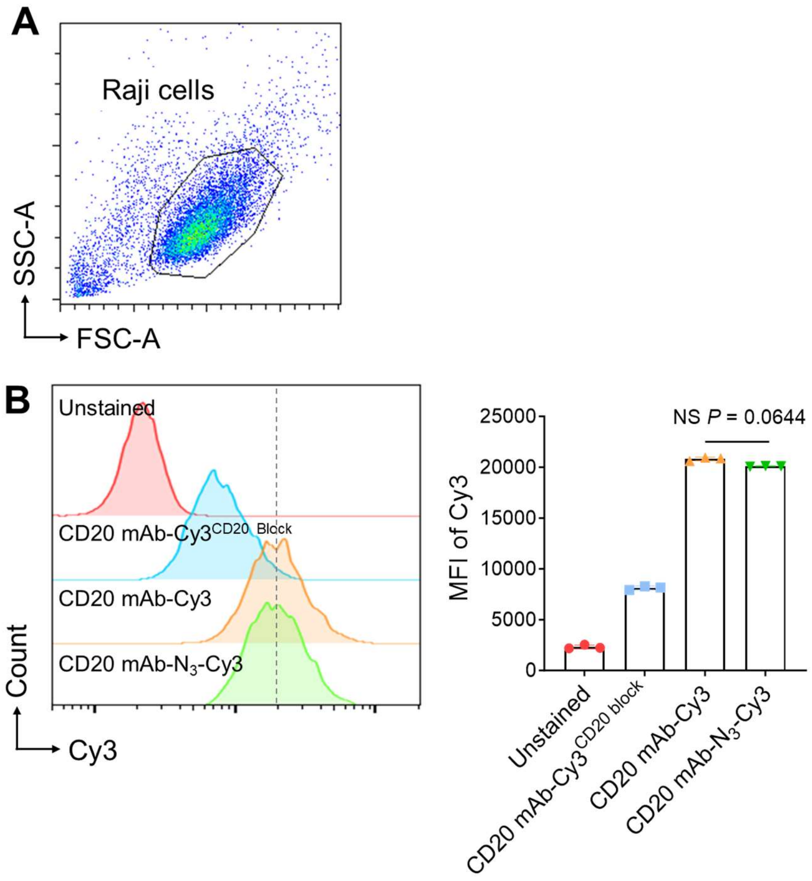

Fig. S2. Flow cytometry analysis of anti-CD20 mAb-N_3 anchoring on the surface of Raji cells. (**A**) Gating strategy for Raji cells. (**B**) Fluorescence intensity of anti-CD20 mAb-N_3 anchoring on Raji cells. Unstained, Raji cells (2×10^5) in culture medium; CD20 mAb-Cy3$^{CD20\ block}$, Raji cells were pre-blocking by 10 µg anti-CD20 mAb at 37°C for 1 h, then 1 µM anti-CD20 mAb-N_3-Cy3 was added and incubated at 37°C for 1 h; CD20 mAb-Cy3, Raji cells were treated with 1 µM anti-CD20 mAb-Cy3 at 37°C for 1 h; CD20 mAb-N_3-Cy3, Raji cells were treated with 1 µM anti-CD20 mAb-N_3-Cy3 at 37°C for 1 h. The cells were analyzed by flow cytometry. Data are presented as mean ± SD (n = 3). Statistics are calculated via one way ANOVA followed by Tukey's multiple comparisons. NS not significant.

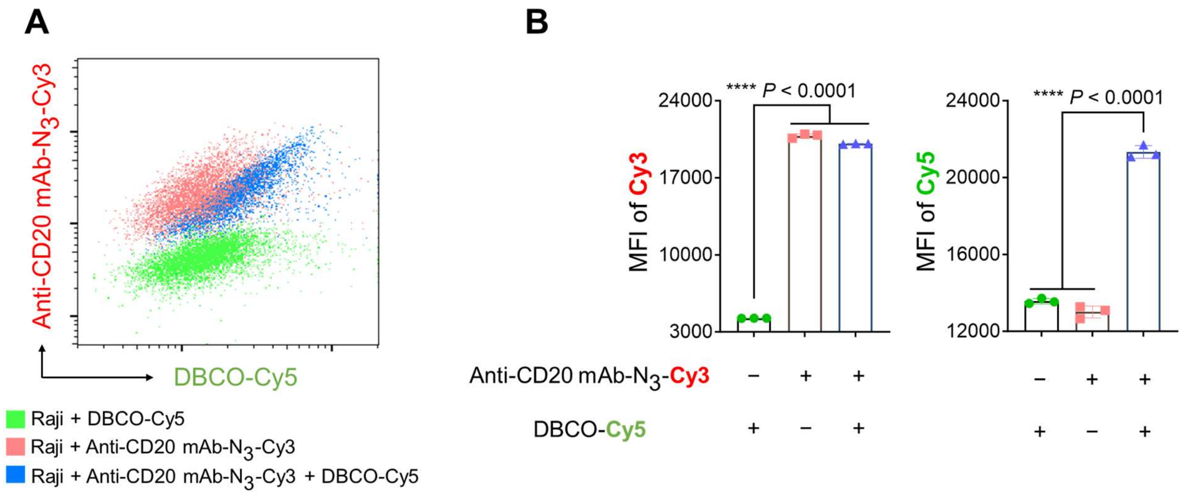

Fig. S3. Flow cytometry analysis confirming surface exposure of clickable azide groups by the bifunctional adaptor. (**A**) Representative scatter plots. (**B**) Fluorescence intensity of anti-CD20 mAb-N_3-Cy3 or DBCO-Cy5. Data are presented as mean ± SD (n = 3). Statistics are calculated via one way ANOVA followed by Tukey's multiple comparisons. ****$P < 0.0001$.

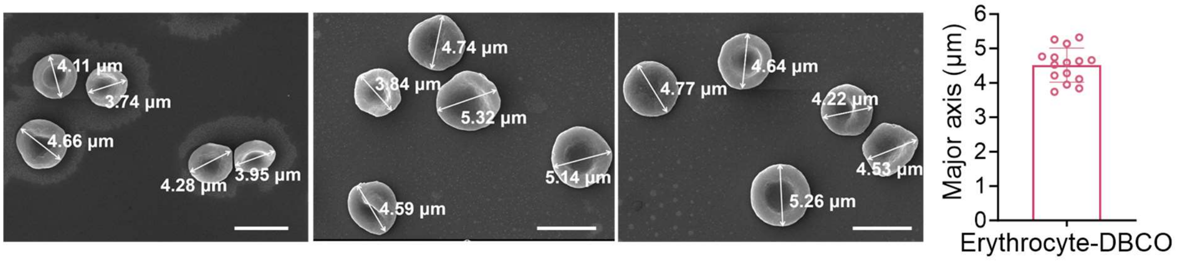

Fig. S4. Average size of initial Erythrocyte-DBCO in CD20 EryPatch. Scale bar, 5 µm. n = 15.

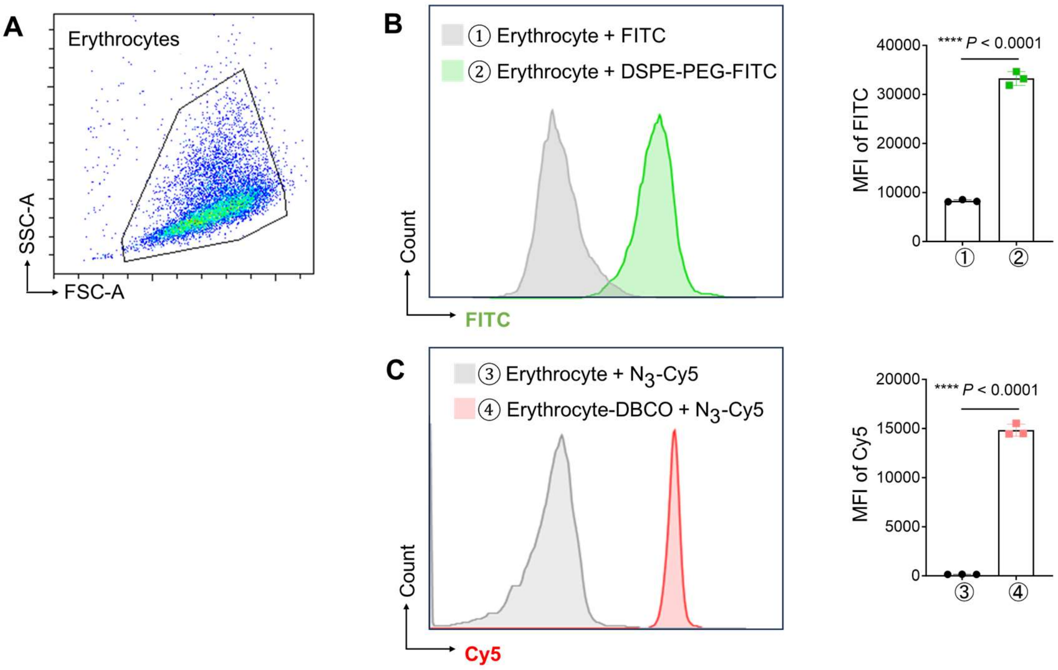

Fig. S5. Stepwise surface functionalization of erythrocytes analyzed by flow cytometry. (**A**) Gating strategy for erythrocyte. (**B**) Membrane insertion of FITC-PEG_{2000}-DSPE. (**C**) Sequential DBCO-PEG_{2000}-DSPE insertion and bioorthogonal click reaction with N_3-Cy5. Data are presented as mean ± SD (n = 3). Statistics are calculated via unpaired Student's t test. ****$P < 0.0001$.

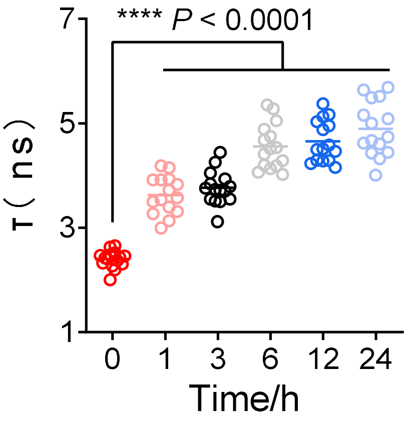

Fig. S6. Quantitative analysis of alterations in surface tension on Erythrocyte-DBCO at predetermined time points after the cell-surface lipid insertion. Data are presented as mean ± SD ($n = 15$). Statistics are calculated via one way ANOVA followed by Tukey's multiple comparisons. ****$P < 0.0001$.

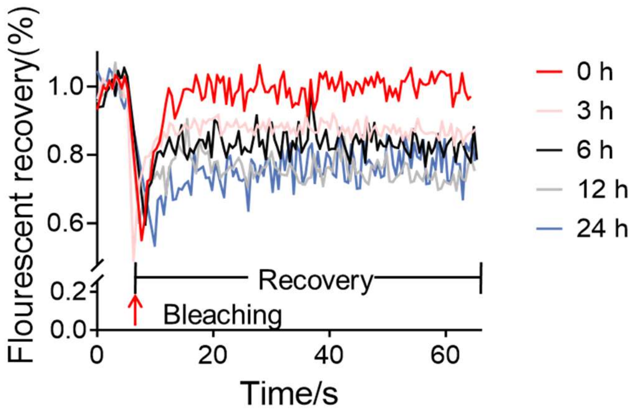

Fig. S7. Fluorescence recovery after photobleaching (FRAP) analysis of erythrocyte membrane fluidity at predetermined time points after DBCO-PEG_{2000}-DSPE insertion. In the FRAP experiment, the membrane of Erythrocyte-DBCO cells were stained with DiD fluorescent dye. The fluorescent molecules in a defined region are photobleached with a high-power laser, and subsequent fluorescence recovery is monitored. Lower recovery rates indicate reduced membrane fluidity.

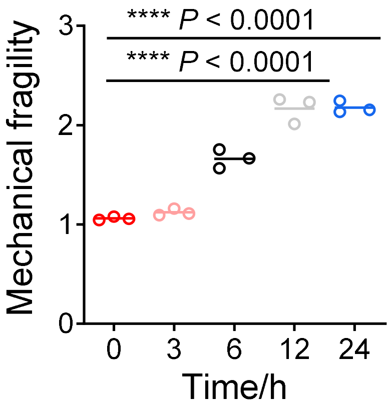

Fig. S8. Mechanical fragility assessed by hemolysis rate at predetermined time points after DBCO-PEG$_{2000}$-DSPE insertion. Data are presented as mean ± SD ($n = 3$). Statistics are calculated via one way ANOVA followed by Tukey's multiple comparisons. ****$P < 0.0001$.

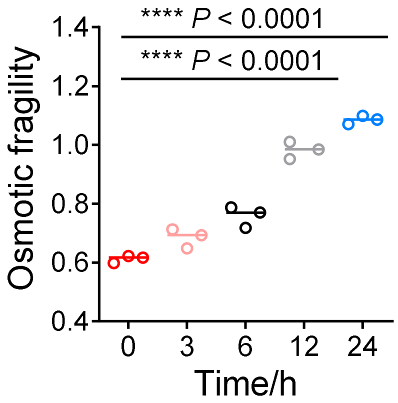

Fig. S9. Osmotic fragility quantified by hemolysis in graded hypotonic saline solutions at predetermined time points after DBCO-PEG_{2000}-DSPE insertion. Data are presented as mean ± SD ($n = 3$). Statistics are calculated via one way ANOVA followed by Tukey's multiple comparisons. ****$P < 0.0001$.

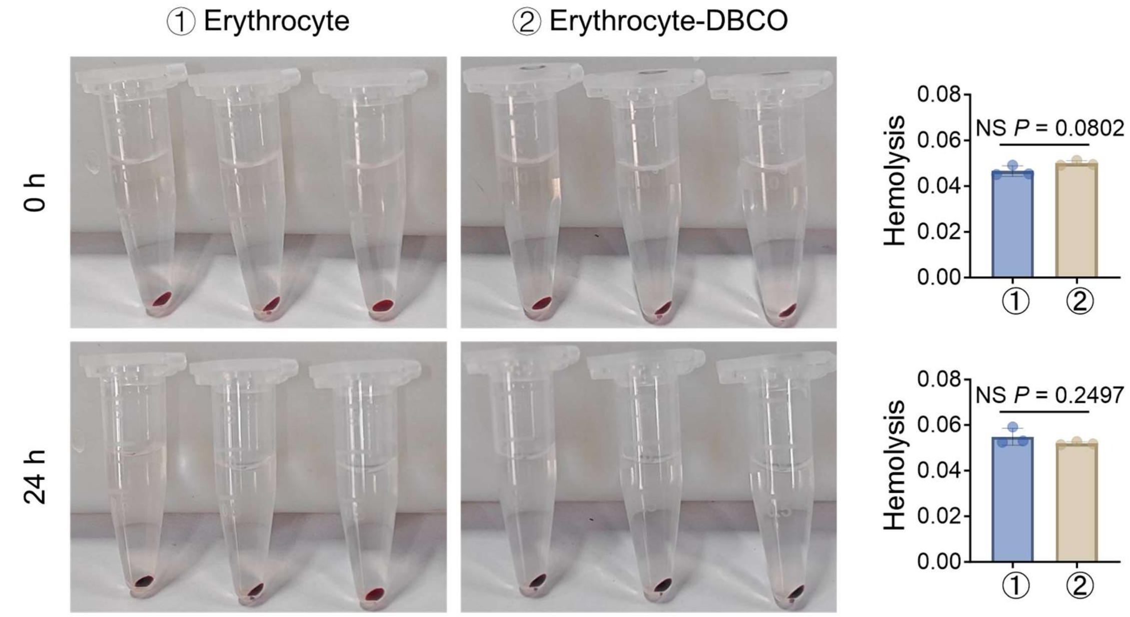

Fig. S10. Hemolysis assessment of Erythrocyte-DBCO after the cell-surface lipid insertion. OD545 values of supernatants from erythrocytes and Erythrocyte-DBCO were measured at 0 h and 24 h post lipid insertion to quantify hemolysis levels. Data presented as mean ± SD (n = 3). Data are presented as mean ± SD (n = 3). Statistics are calculated via unpaired Student's t test. NS not significant.

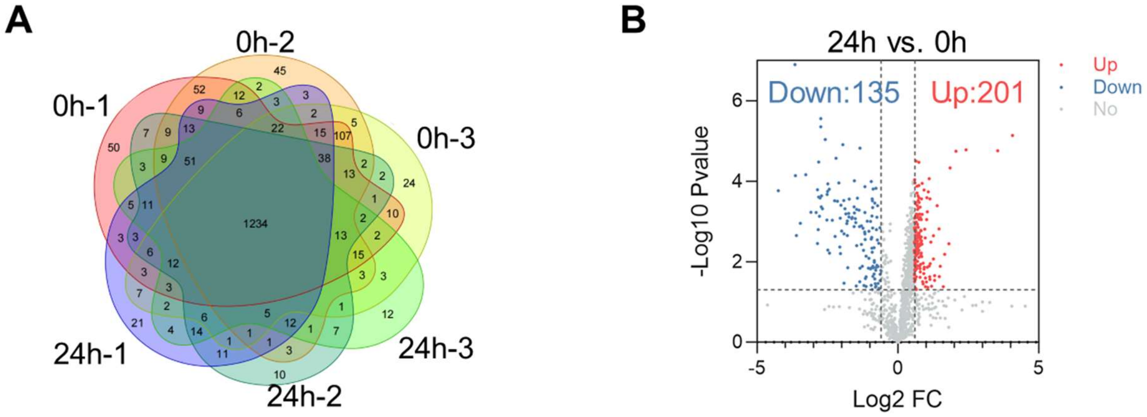

Fig. S11. Proteomics analyses of Erythrocyte-DBCO 24 h post-insertion of DBCO-PEG$_{2000}$-DSPE. (**A**) Veen diagram of DEPs depicting the number of shared and unique proteins in each group. (**B**) Volcano plot exhibiting significantly up/down regulated proteins identified through multiple comparison.

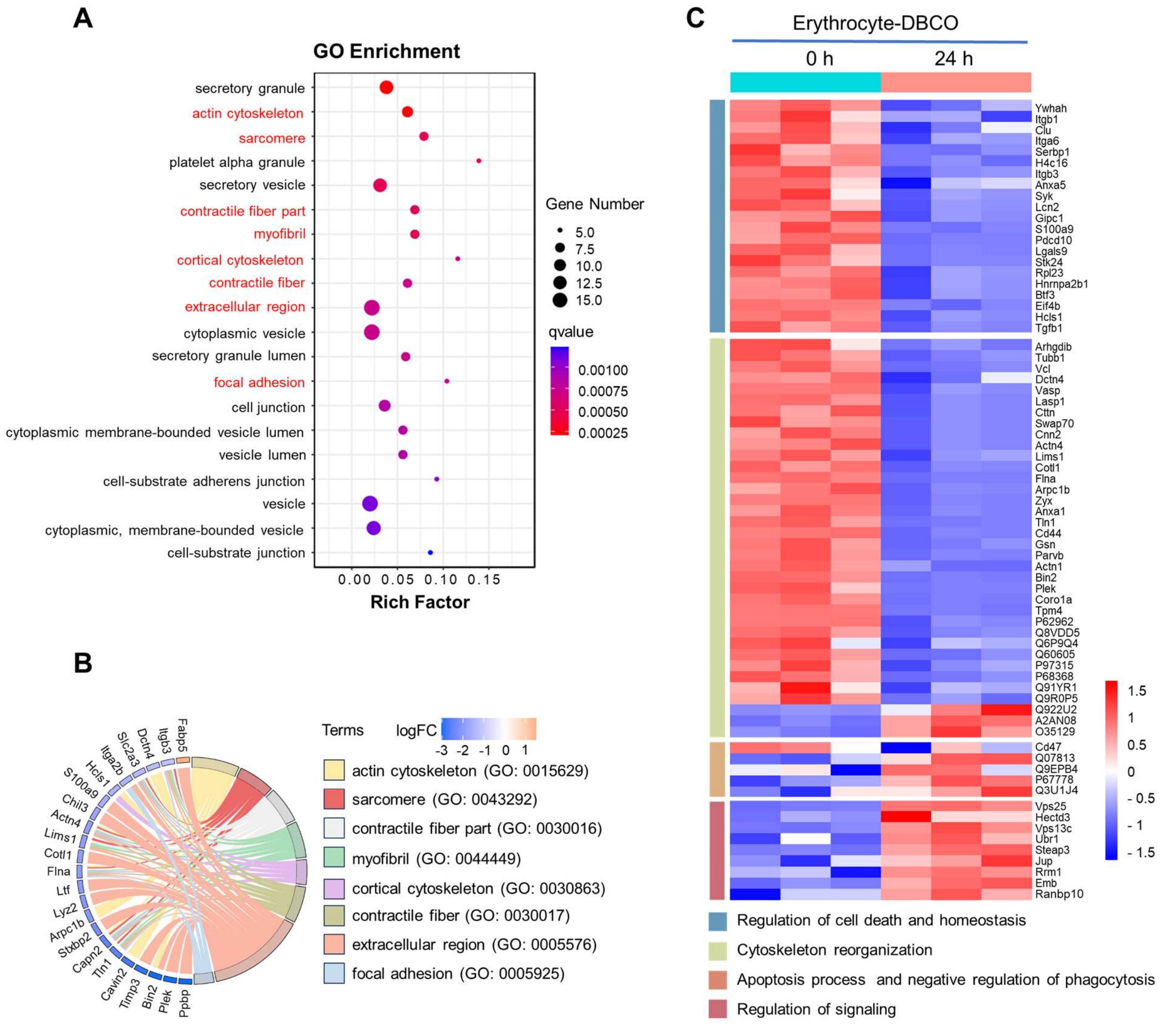

Fig. S12. Proteomics analyses of Erythrocyte-DBCO 24 h post-insertion of DBCO-PEG$_{2000}$-DSPE. (**A**) enrichment analysis of Gene Ontology (GO) terms of DEPs performed via one-way comparison. (**B**) chord diagram display of GO terms. (**C**) heat map display of selected proteins involved in biological signaling and structural remodeling.

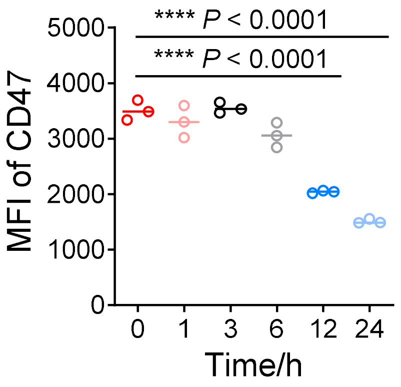

Fig. S13. Flow cytometry analysis of the eventual alteration in CD47 expression on Erythrocyte-DBCO at predetermined time points after the cell-surface lipid insertion. Data are presented as mean ± SD ($n = 3$). Statistics are calculated via one way ANOVA followed by Tukey's multiple comparisons. ****$P < 0.0001$.

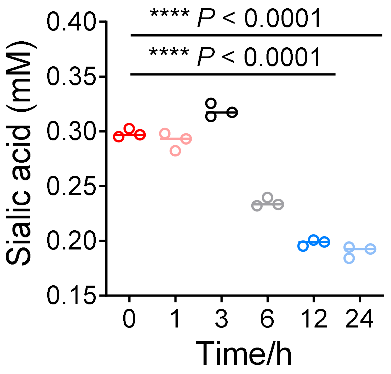

Fig. S14. Quantification of sialic acid on Erythrocyte-DBCO at predetermined time points after the cell-surface lipid insertion. Data are presented as mean ± SD (n = 3). Statistics are calculated via one way ANOVA followed by Tukey's multiple comparisons. ****$P < 0.0001$.

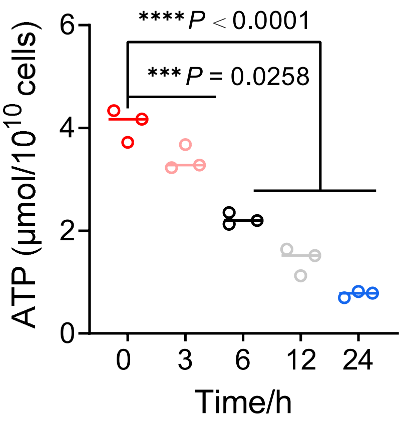

Fig. S15. Time-course of ATP production by Erythrocyte-DBCO after the cell-surface lipid insertion over a 24 h period. Data are presented as mean ± SD ($n = 3$). Statistics are calculated via one way ANOVA followed by Tukey's multiple comparisons. ***$P < 0.001$, ****$P < 0.0001$.

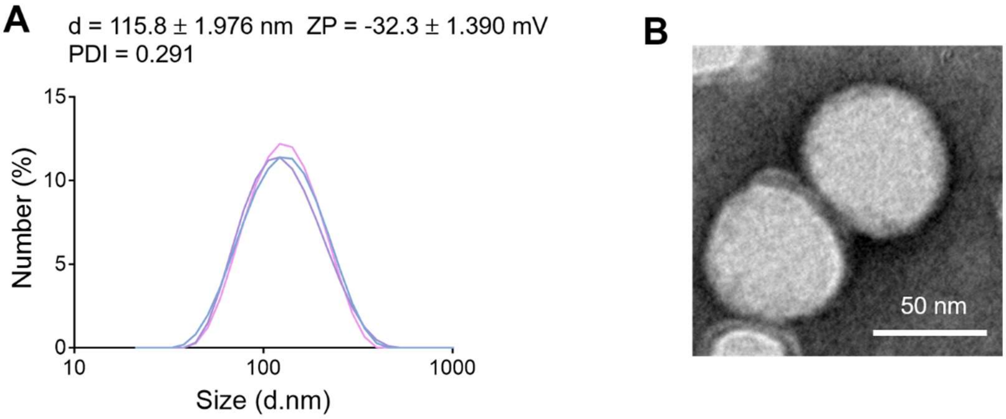

Fig. S16. Characterization of DBCO-NP. (**A**) Size distribution. (**B**) Transmission electron microscopy images, Scale bar, 50 nm.

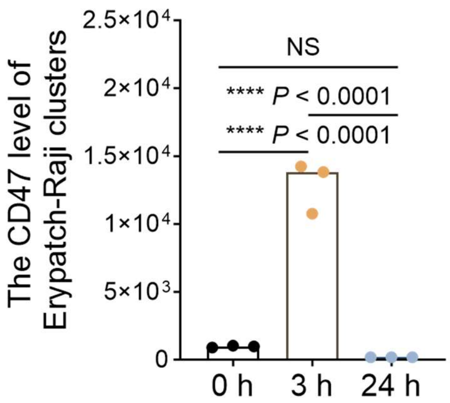

Fig. S17. Quantification of CD47 fluorescence intensity gated within GFP^+ cell clusters. Data are presented as mean ± SD ($n = 3$). Statistics are calculated via one way ANOVA followed by Tukey's multiple comparisons. NS not significant, ****$P < 0.0001$.

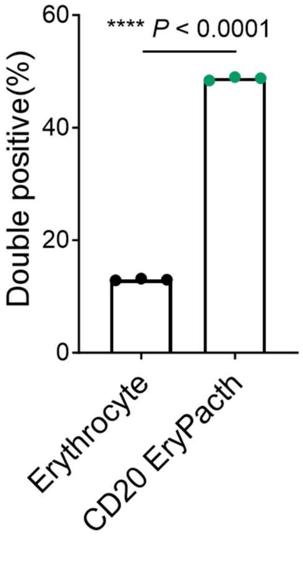

Fig. S18. Quantification of phagocytosis of Raji-GFP cells by DiD-labeled BMDMs (BMDM-DiD). Data are presented as mean ± SD (n = 3). Statistical significance was calculated via unpaired Student's t test. ****$P < 0.0001$.

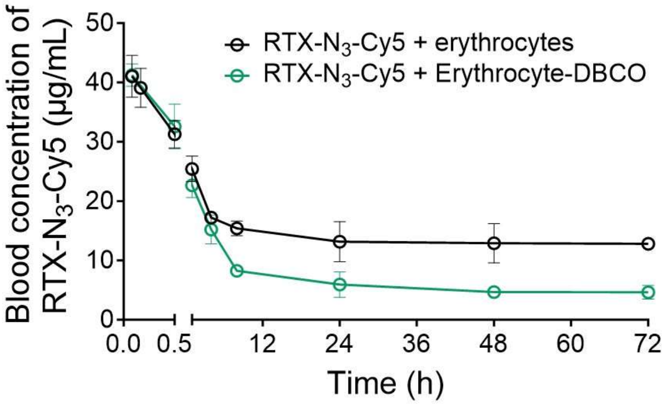

Fig. S19. The blood concentration-time profile of Cy5-labeled anti-CD20 mAb-N_3 (RTX-N_3-Cy5) in healthy CB-17 SCID mice. Randomly divided mouse groups received intravenous administration of RTX-N_3-Cy5 (1 nmol), followed by injection 5 h later with erythrocytes (1 × 10^7 cells) or Erythrocyte-DBCO (1 × 10^7 cells). Data points represent the mean blood antibody concentration ± SD (n = 5), quantified by converting Cy5 fluorescence intensity using a standard curve and expressed in micrograms per milliliter (μg/mL).

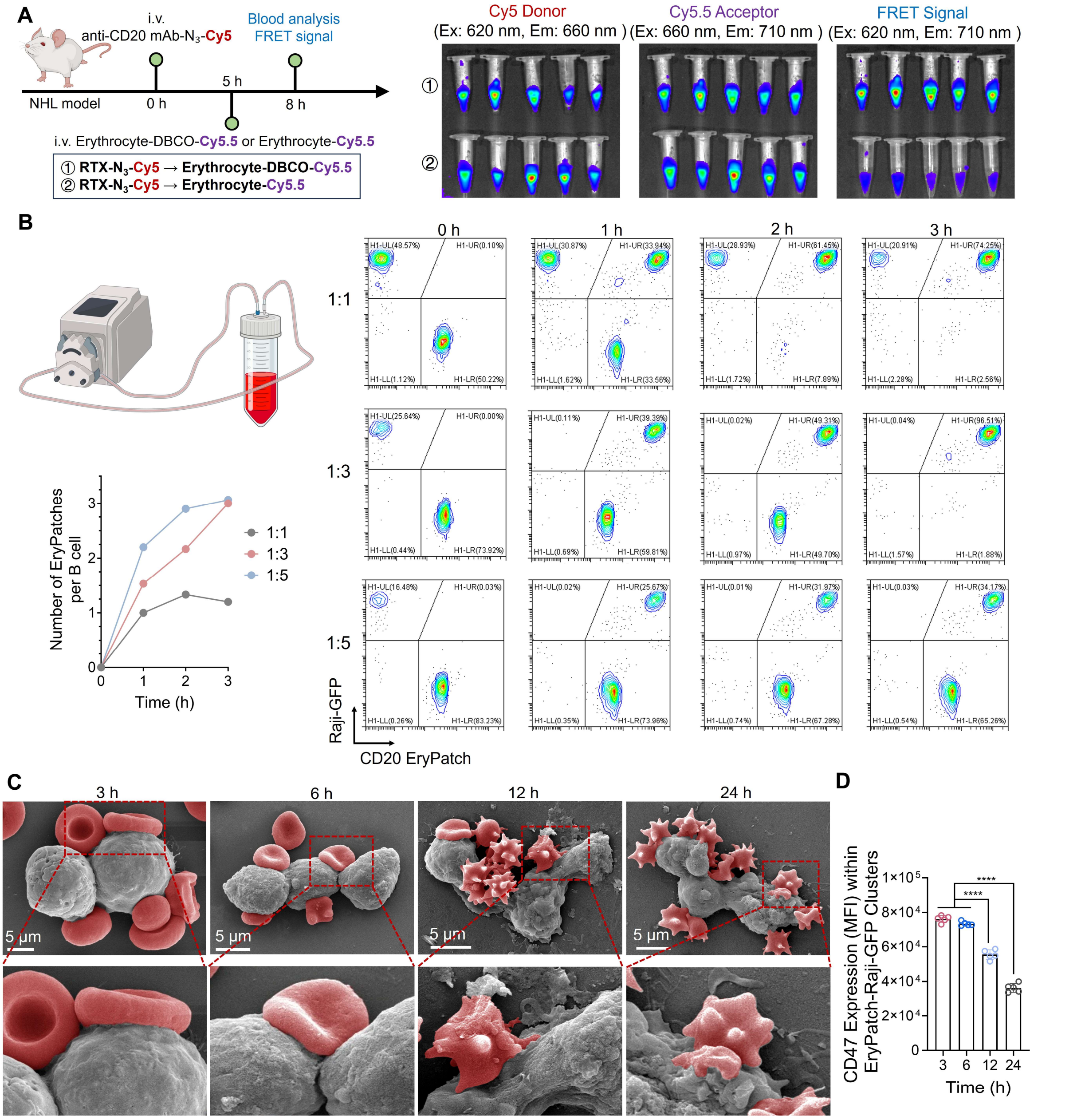

Fig. S20. CD20 EryPatch binding kinetics and stabilities during circulation. (**A**) FRET analysis of blood samples collected from NHL mouse model receiving consecutive treatment (RTX-N_3-Cy5 → Erythrocyte-DBCO-Cy5.5, or RTX-N_3-Cy5 → Erythrocyte-Cy5.5) (n = 5). (**B**) Quantitative CD20 EryPatch-B cell binding kinetics analysis using a blood-circulating mimetic device. RTX-N_3-pre-targeted Raji-GFP cells were mixed with red-fluorescent Erythrocyte-DBCO at varying ratios and circulated for indicated time points (1, 2, 3 h), prior to flow cytometry analysis with subsequent calculation of EryPatches per B cell (n = 3). (**C** and **D**) Representative SEM images depicting a progressive discocyte-to-echinocyte transition of CD20 EryPatch on Raji B cells (C) and flow cytometry analysis of the time-dependent CD47 loss within CD20 EryPatch-B cell clusters (D) after RTX-N_3-pre-targeted Raji-GFP cells were mixed with Erythrocyte-DBCO

at 1:3 ratio and circulated for indicated time points (3, 6, 12, 24 h) in the blood-circulating mimetic device. Grey indicates Raji B-cells, Red indicates CD20 EryPatch. Scale bar, 5 μm. Data are presented as mean ± SD ($n = 5$). Statistics are calculated via one way ANOVA followed by Tukey's multiple comparisons. ****$P < 0.0001$.

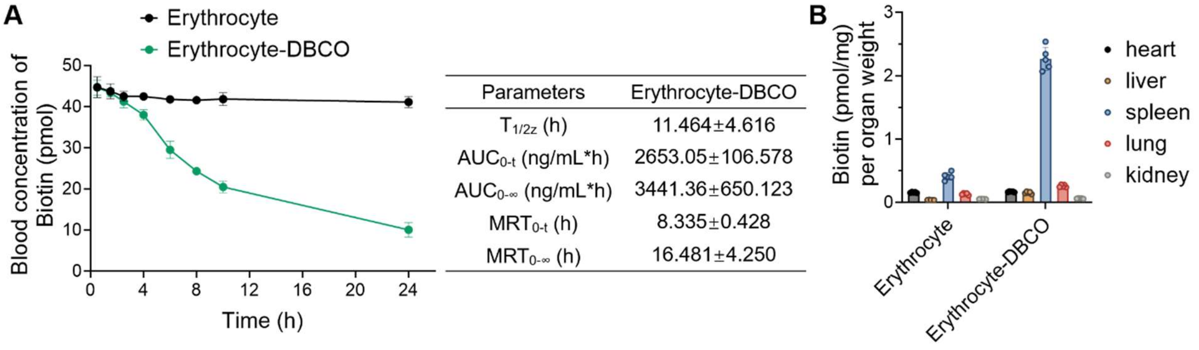

Parameters	Erythrocyte-DBCO
$T_{1/2z}$ (h)	11.464±4.616
$AUC_{0\text{-}t}$ (ng/mL*h)	2653.05±106.578
$AUC_{0\text{-}\infty}$ (ng/mL*h)	3441.36±650.123
$MRT_{0\text{-}t}$ (h)	8.335±0.428
$MRT_{0\text{-}\infty}$ (h)	16.481±4.250

Fig. S21. Pharmacokinetics and biodistribution of Erythrocyte-DBCO. (**A**) Pharmacokinetic profile after intravenous administration of biotinylated Erythrocyte-DBCO in healthy CB-17 SCID mice. Blood was collected at different time points, and biotin concentration was quantified by ELISA. Pharmacokinetic parameters of statistical moment analysis are calculated using DAS 2.0 software. $T_{1/2z}$: half-life, AUC: area under curve, MRT: mean residence time. (**B**) Biodistribution at 24 h post-injection. Major organs (heart, liver, spleen, lung, kidney) were homogenized, and biotin accumulation was measured per organ weight. Data are presented as mean ± SD (n = 5).

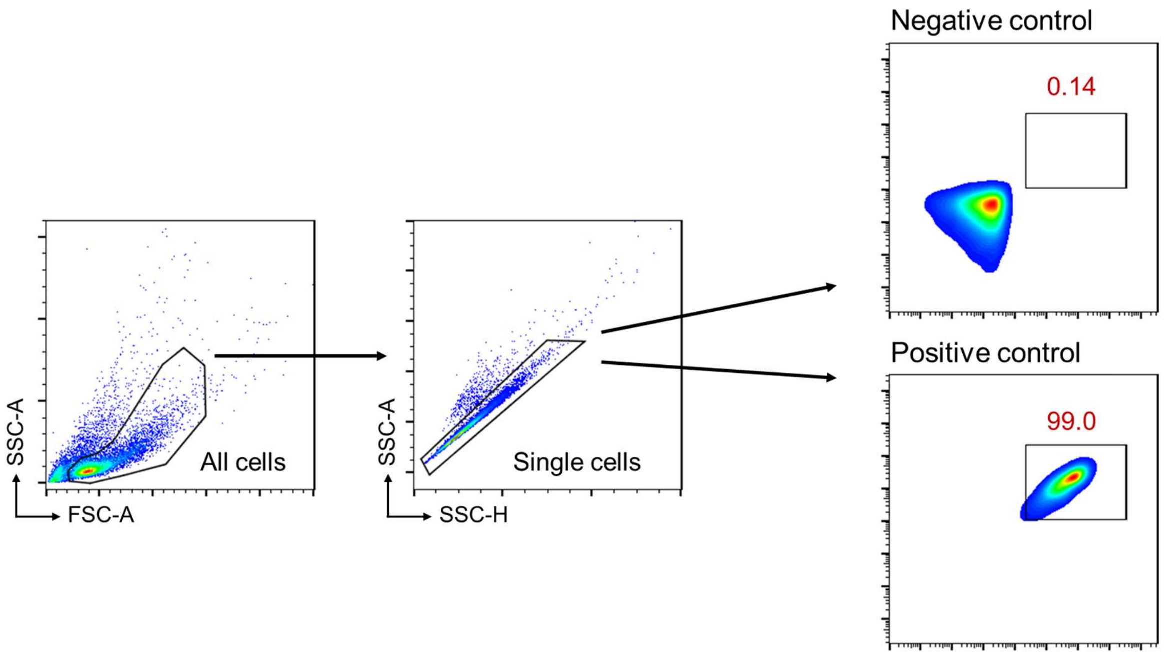

Fig. S22. Gating strategy and flow cytometry analysis of residual Raji cells (human $CD10^{+}CD19^{+}$) in the bone marrow (BM). BM cells isolated from native CB-17 SCID mice served as the negative control, and Raji cells served as the positive control.

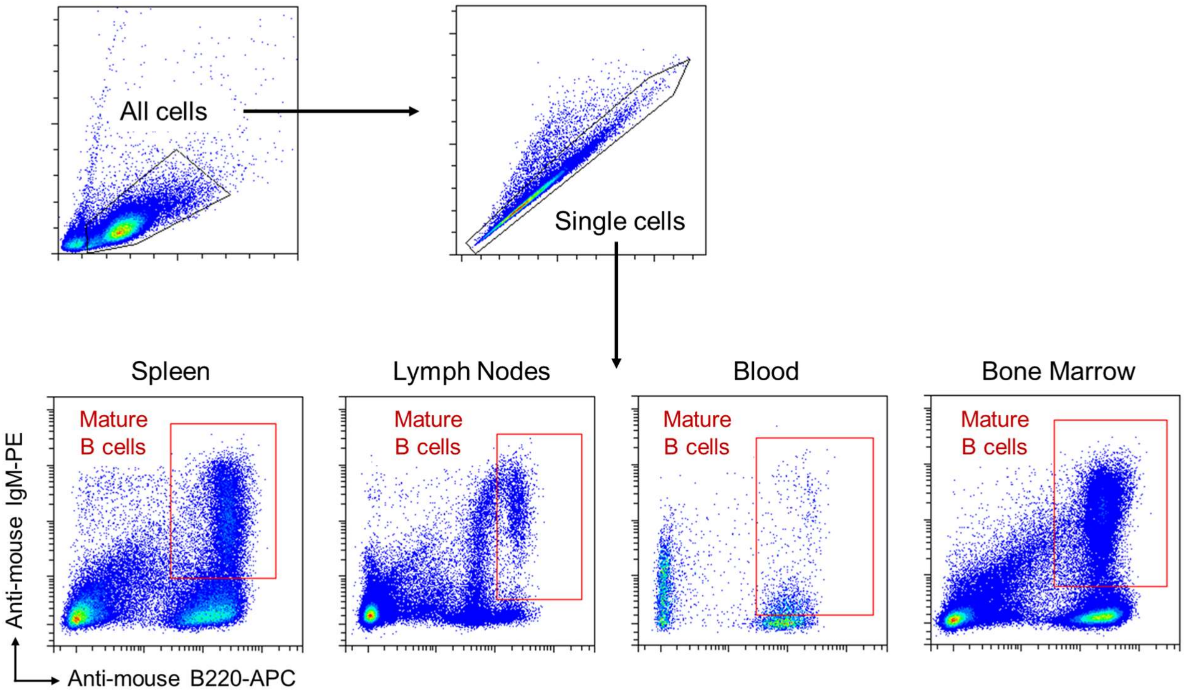

Fig. S23. Gating strategy and flow cytometry analysis of mature B cells ($IgM^{+}B220^{high}$) across the spleen, lymph nodes, blood, and bone marrow.

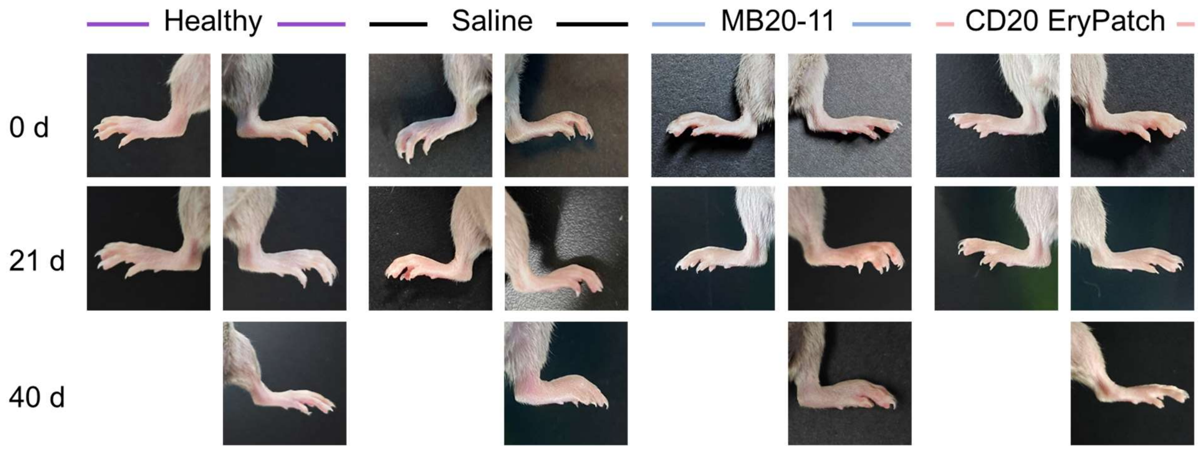

Fig. S24. Representative images show the condition of hind paws with different treatments at predetermined time points. The left paw images on 40 d are displayed in Fig. 6H.

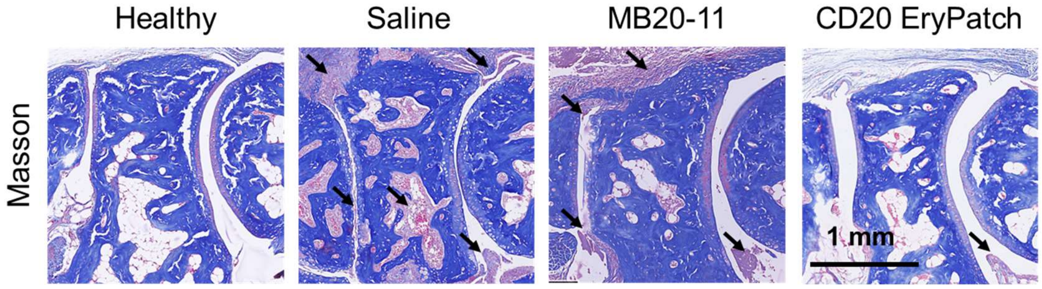

Fig. S25. Representative histology of ankle joint sections stained with Masson's trichrome staining with different treatments. Scale bar, 1 mm.

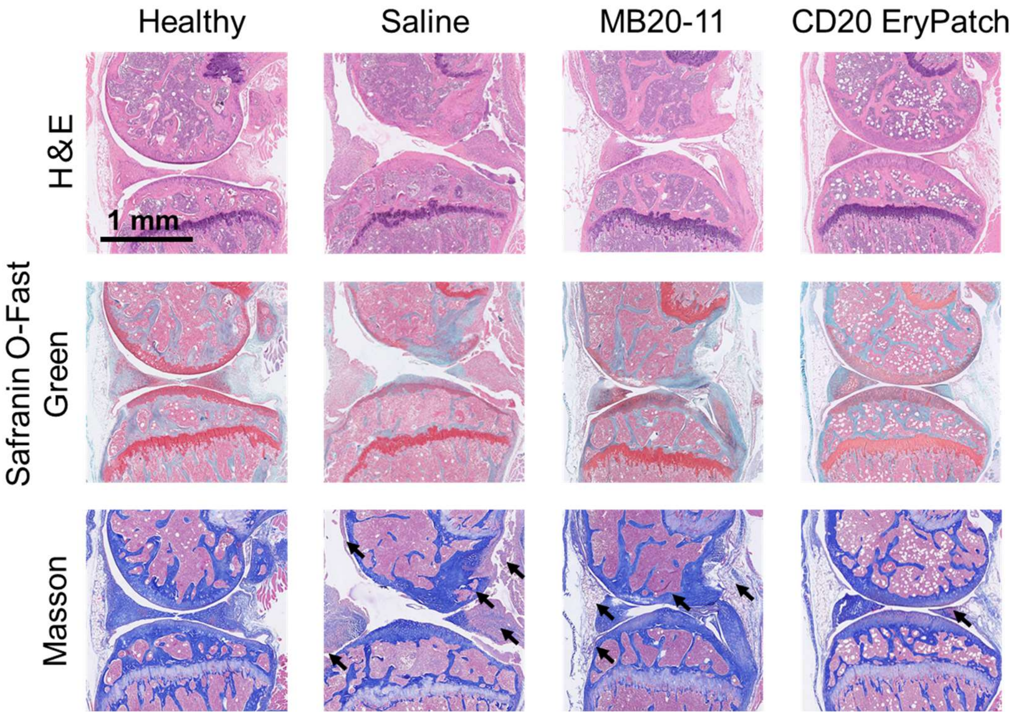

Fig. S26. Representative histology of knee joint sections stained with H&E, Safranin O-Fast green, and Masson's trichrome staining with different treatments. Scale bar, 1 mm.

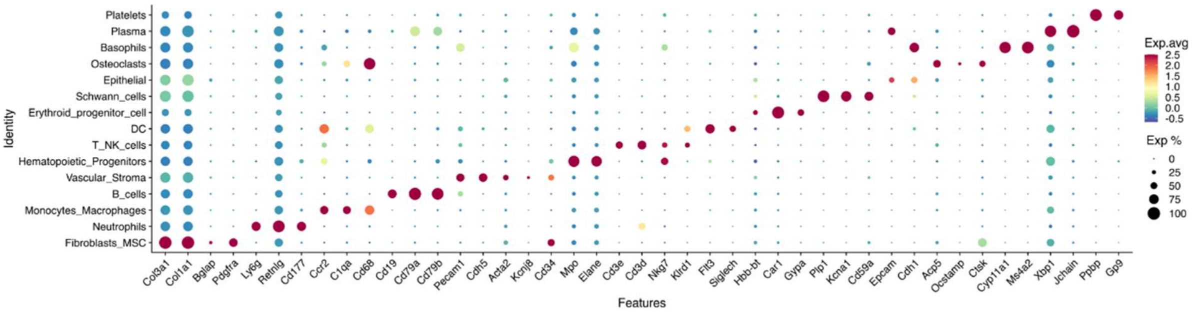

Fig. S27. Bubble heatmap showed the marker genes of the defined 15 clusters.

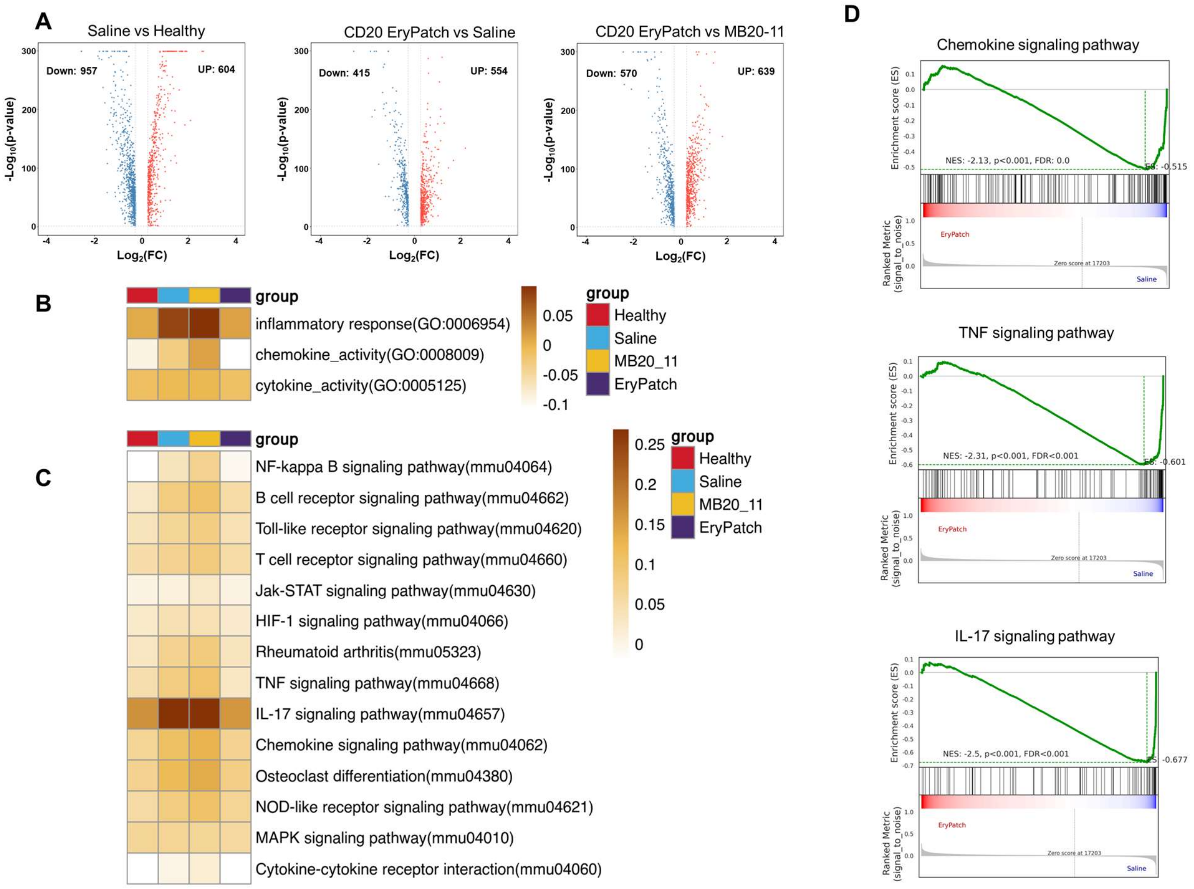

Fig. S28. The analysis of inflammatory pathways from different groups. (**A**) Volcano plots of differentially expressed genes comparing Saline versus Healthy, CD20 EryPatch versus Saline, and CD20 EryPatch versus MB20-11. (**B**) Heatmap illustrating the average expression of inflammation-related pathways of GO enrichment from different groups. (**C**) Heatmap illustrating the average expression of inflammation-related pathways of KEGG enrichment from different groups. (**D**) Gene set enrichment analysis (GSEA) pathways from different groups. p-values were calculated by the one-sided Permutation test.

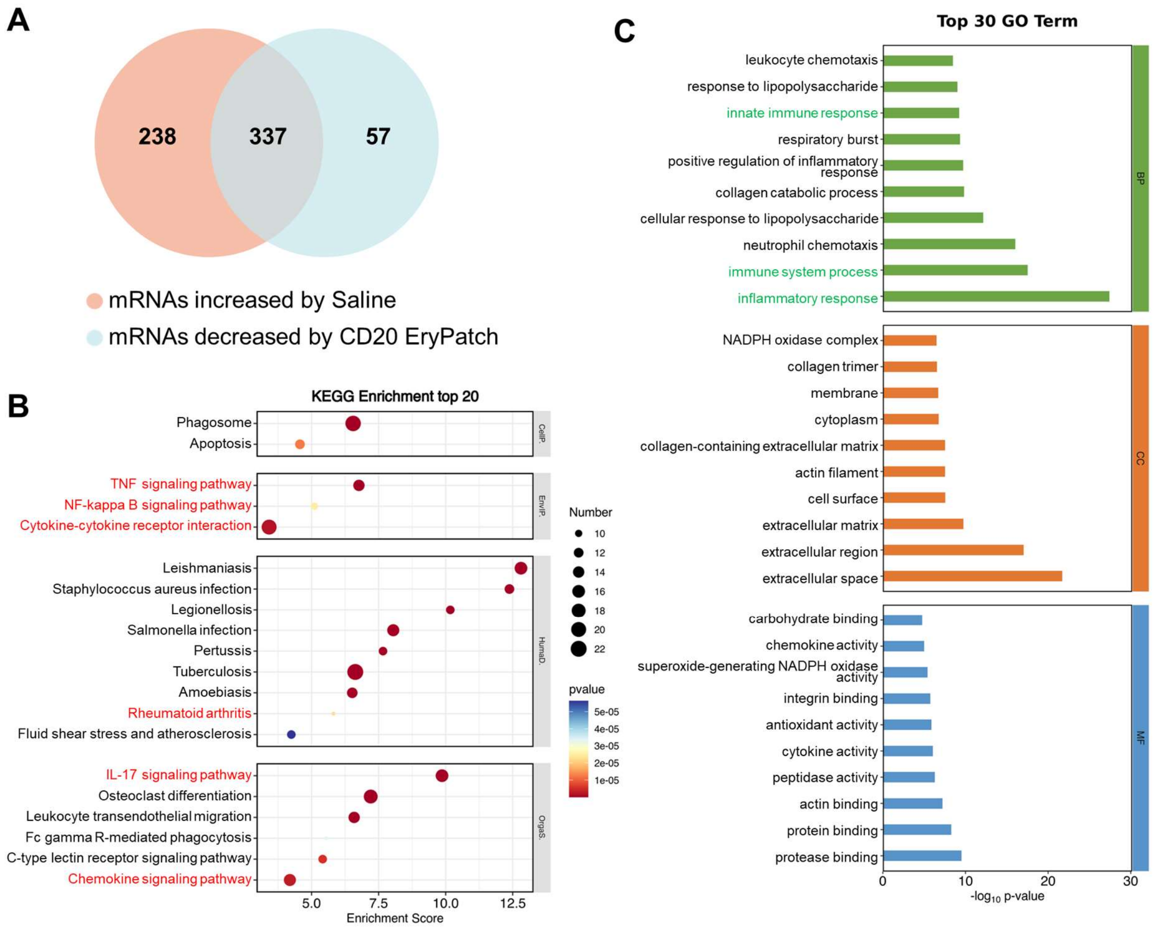

Fig. S29. CD20 EryPatch treatment can alleviate inflammatory responses compared to Saline treatment. (**A**) Venn diagram showing the intersection of genes regulated by Saline and CD20 EryPatch. (**B**) KEGG enrichment analysis of 337 mRNAs that are simultaneously up-regulated by Saline and down-regulated by CD20 EryPatch. (**C**) GO enrichment analysis revealing biological pathways enriched for 337 mRNAs.

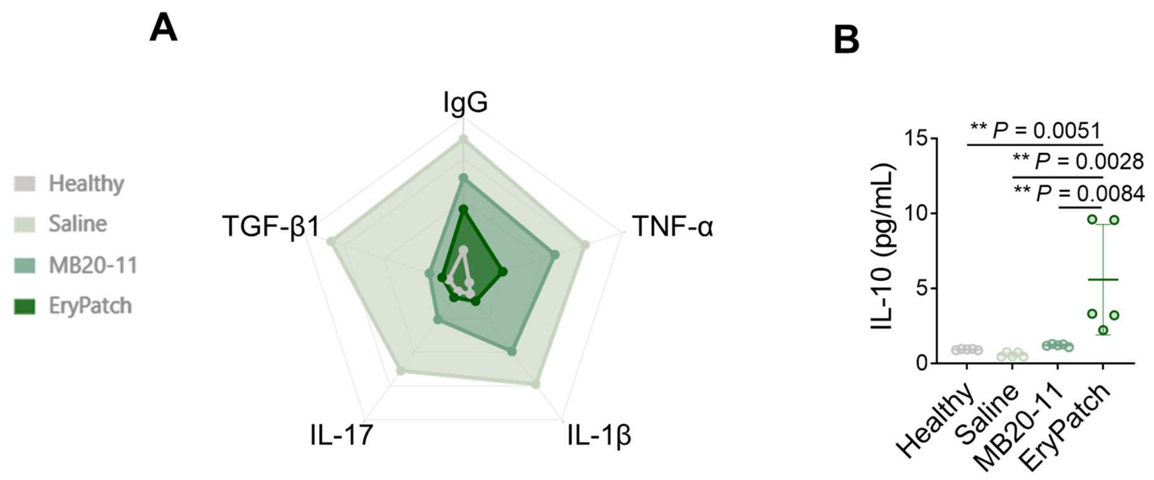

Fig. S30. CD20 EryPatch treatment can reduce the secretion of inflammatory factors. The levels of IgG, TGF-β1, TNF-α, IL-17, IL-1β (**A**), and IL-10 (**B**) in serum measured by ELISA. Data are presented as mean ± SD (n = 3). Statistics are calculated via one way ANOVA followed by Tukey's multiple comparisons. $**P < 0.01$.

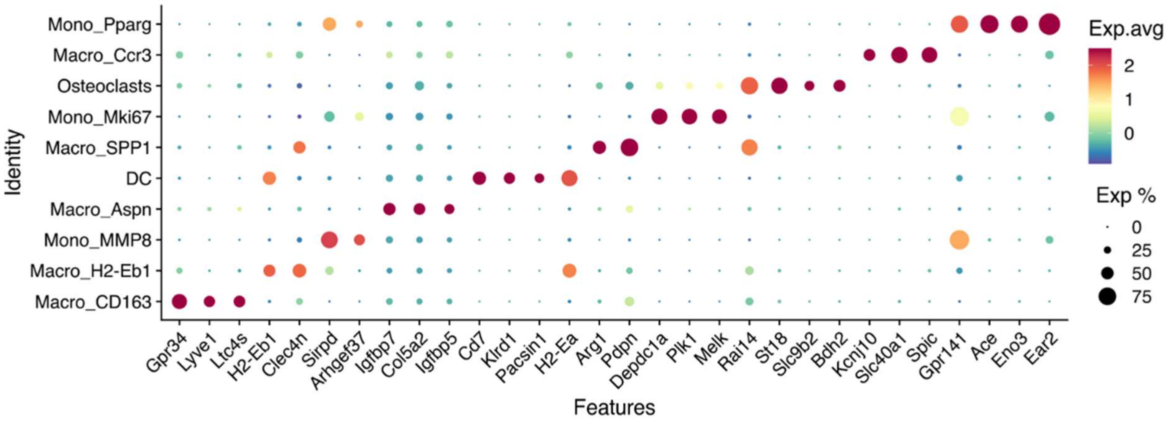

Fig. S31. Bubble heatmap showed the marker genes for each monocyte-macrophages subtype.

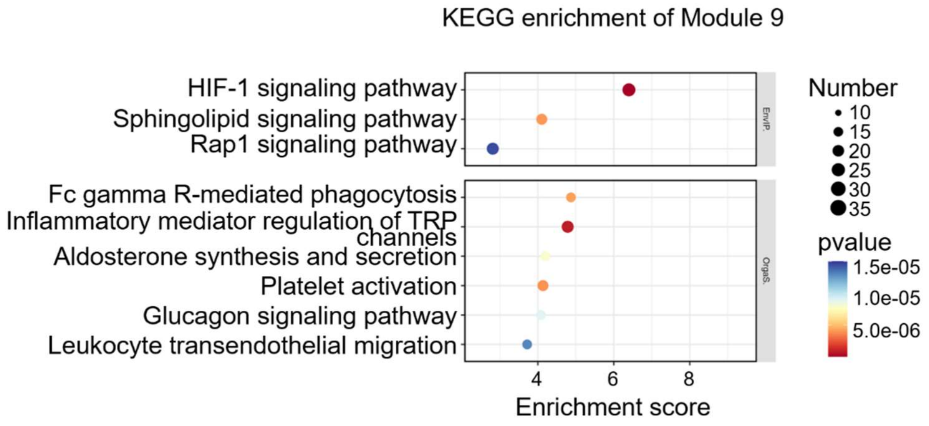

Fig. S32. KEGG analysis revealed an enrichment in immune and inflammatory related signaling pathways within module 9.

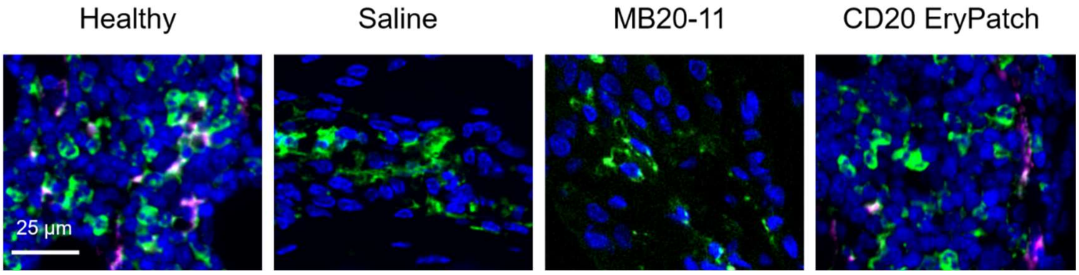

Fig. S33. Representative immunofluorescence staining of knee joint sections for the macrophages ($F4/80^+$) and M2 anti-inflammatory macrophages ($F4/80^+CD206^+$). Green indicates F4/80, pink indicates CD206, blue indicates cell nuclei. Scale bars, 25 μm.

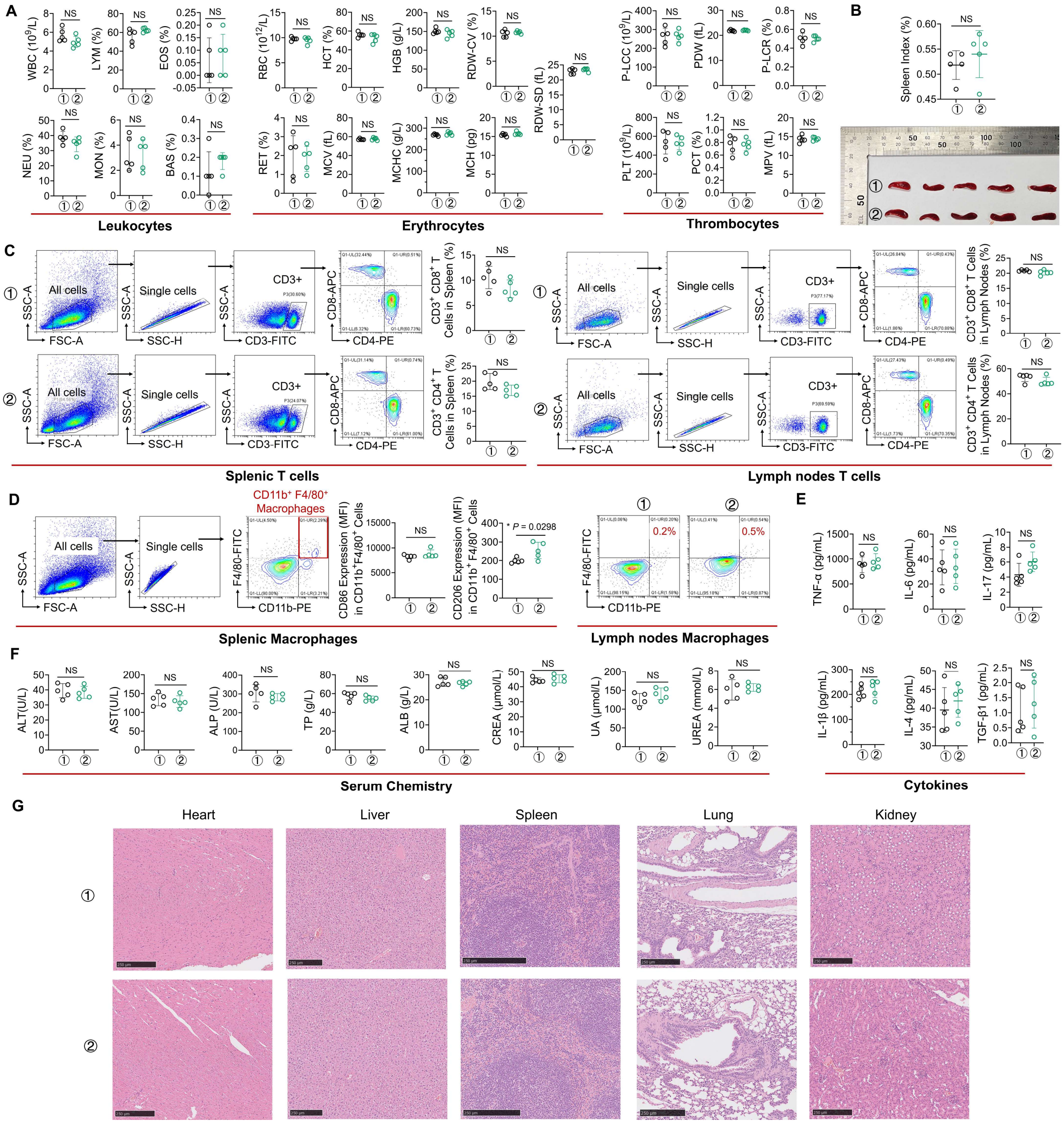

Fig. S34. Biosafety evaluation. Healthy, immunocompetent DBA/1J mice received CD20 EryPatch (MB20-11-N_3, 1 nmol → Erythrocyte-DBCO, 1×10^7erythrocytes) on Day 0, 2, and 4, followed by biosafety analysis on Day 10 (n = 5). Treatment groups: ① Untreated; ② CD20 EryPatch. (**A**) Complete blood count (CBC) analyses. Leukocytes: WBC, white blood cell; LYM, lymphocyte; EOS, eosinophil; NEU, neutrophil; MON, monocyte; BAS, basophil; Erythrocytes: RBC, red blood cell; HCT, hematocrit value; HGB, hemoglobin; RDW, red blood cell volume distribution width; RET, reticulocyte; MCV, mean corpuscular volume; MCHC, mean corpuscular hemoglobin concentration; MCH, mean corpuscular hemoglobin; Thrombocytes: P-LCC, platelet-large cell count; PDW, platelet volume distribution width; P-LCR, platelet-large cell ratio; PLT, platelet; PCT, plateletcrit; MPV, mean platelet volume. (**B**) Extent of splenomegaly, shown by representative images and organ weight indices for spleens. (**C**) Quantitative flow

cytometry analysis of $CD3^+CD4^+$ T cells and $CD3^+CD8^+$ T cells frequencies in spleen and lymph nodes. (**D**) Quantitative flow cytometry analysis of $CD11b^+F4/80^+$ macrophages with CD86 and CD206 expressions in spleen and lymph nodes. **(E)** Measurement of key pro-inflammatory (TNF-α, IL-6, IL-17, IL-1β) or regulatory (IL-4, TGF-β) cytokine levels in the serum. (**F**) Serum chemistry analysis. ALT, alanine aminotransferase; AST, aspartate aminotransferase; ALP, alkaline phosphatase; TP, total protein; ALB, albumin; CREA, creatinine; UA, uric acid. (**G**) Representative hematoxylin and eosin staining images of major organs (heart, liver, spleen, lung, kidney). Scale bar, 250 μm. Data are presented as mean ± SD. Statistical significance in (A-F) was calculated via unpaired Student's *t* test. NS not significant, $*P < 0.05$.

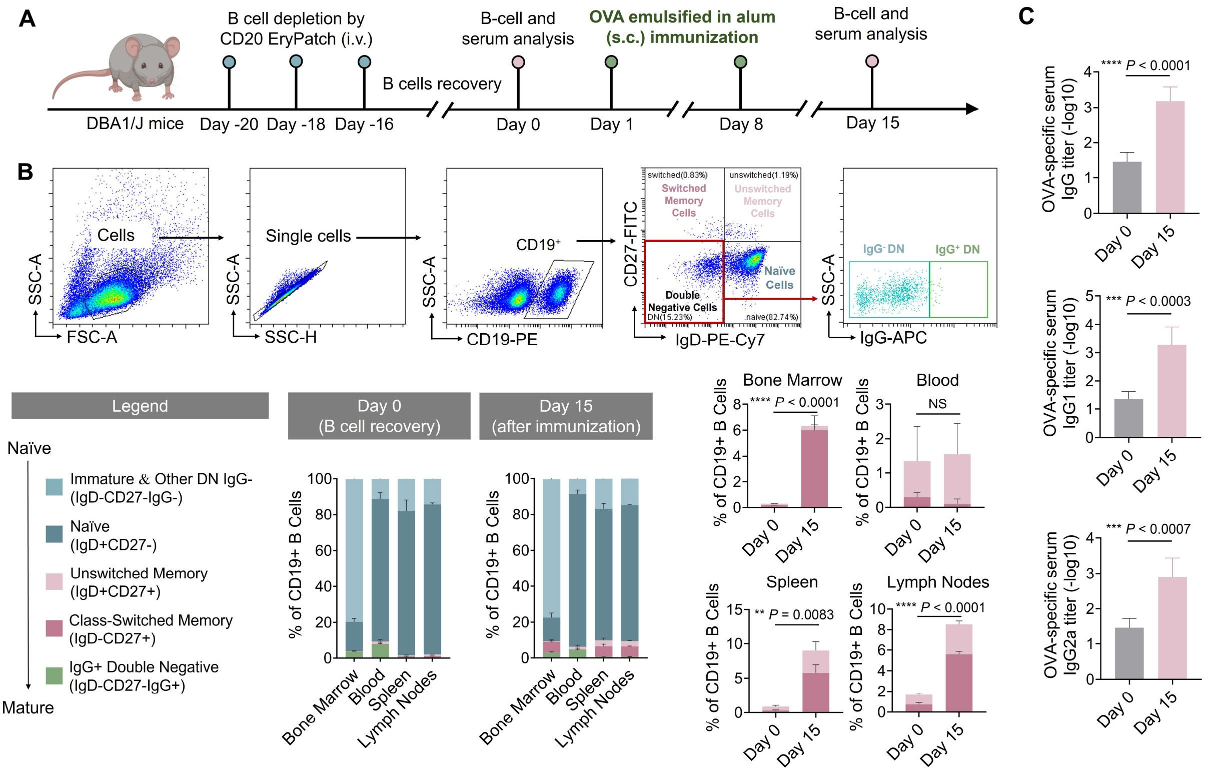

Fig. S35. Repopulated B cell response to immunization after CD20 EryPatch therapy terminated. (**A**) DBA1/J mice receiving three doses of CD20 EryPatch were allowed 16 days for B cell recovery. Mice were then immunized subcutaneously with OVA emulsified in alum on Day 1, boosted on Day 8, and analyzed on Day 15. (**B**) Flow cytometry gating strategy and enumeration of B-cell subsets in bone marrow, blood, spleen and lymph nodes. DN, double negative; IgG, immunoglobulin G; IgD, immunoglobulin D. (**C**) Serum antibody-titers of anti-OVA IgG, IgG1, and IgG2a antibodies. n = 5 animals per group. Data are presented as mean ± SD. Statistical significance in (B, C) was calculated via unpaired Student's t test. NS not significant, *$P < 0.05$, **$P < 0.01$, ***$P < 0.001$, ****$P < 0.0001$.
